# FFT-GA-BC*:* a fused federated transformer genetic algorithm framework with blockchain-secured, differentially private, homomorphically encrypted aggregation for mutation, phenotype, and binary-allele analysis

**DOI:** 10.3389/fmed.2026.1959427

**Published:** 2026-10-08

**Authors:** Muhammad Umar Nasir, Urva Rehmat, Abdul Khader Jilani Saudagar, Mona Alkhattabi, Muhammad Adnan Khan, Muhammad Amir Khan

**Affiliations:** 1 School of Computing, University of Portsmouth, Portsmouth, United Kingdom; 2 School of Computing, Arden University, London, United Kingdom; 3 School of Computing and Emerging Technologies, Roots IVY College of Management Sciences, Lahore, Pakistan; 4 Faculty of Computing, Riphah International University, Islamabad, Pakistan; 5 Department of Computing, Government Graduate College Women, Hasilpur, Pakistan; 6 Information Systems Department, College of Computer and Information Sciences, Imam Mohammad Ibn Saud Islamic University (IMSIU), Riyadh, Saudi Arabia; 7 Department of Software, Faculty of Artificial Intelligence and Software, Gachon University, Seongnam-si, Republic of Korea; 8 Faculty of Computer and Mathematical Sciences, Universiti Teknologi MARA, Shah Alam, Selangor, Malaysia

**Keywords:** blockchain, clinical genetics, differential privacy, explainability, federated learning, genetic algorithm, homomorphic encryption, multi-hospital pipeline

## Abstract

Federated learning enables cross-institutional clinical-genetics analytics without sharing raw patient data; yet, no existing framework integrates the full cryptographic stack–differential privacy, homomorphic encryption, and blockchain auditing—required for regulatory deployment. Here, we present FFT-GA-BC, a secure-by-construction pipeline combining a bit-flip genetic algorithm for binary feature selection across 136-dimensional genomic representations, a multi-head self-attention encoder, population-stratified federated averaging, (*ε*,*δ*)-differential privacy via the analytic Gaussian mechanism, Cheon–Kim–Kim–Song homomorphic encryption for cipher-domain weight aggregation, and a Practical Byzantine Fault Tolerance (PBFT)-consensus blockchain with SHA-256 Merkle-root commitments per federation round. We evaluate the framework under a simulated multi-institutional deployment scenario using a 950-record synthetic benchmark whose schema mirrors the Punjab Thalassemia and Genetic Disorders Programme Lahore registry, a South Asian clinical-genetics registry operating across six ancestry strata federated across a simulated 12-hospital, five-continent topology, and internally externally validated via a leave-one-population-out proxy across all six ancestry cohorts. Under these conditions, differential privacy imposes less than a 2.7 percentage-point utility loss across the clinically relevant privacy-budget range, with *ε* = 1 recommended as the clinical operating point (Section 5.8), and fully encrypted federated training matches centralised pooled training within ±1.6 percentage points across five independent splits. The blockchain audit layer scales to 192 nodes at 1.3 s of per-round latency with on-chain audit costs below USD 350 per block, maintaining an *O*(log *K*) Merkle-inclusion proof complexity per query. Information-theoretic profiling of the benchmark confirms that labels carry at most 0.036 bits of mutual information against a target entropy of 2.0 bits, demonstrating that the pipeline's diagnostic output faithfully reflects data structure rather than architectural artefacts, a critical validation property for clinical deployment. To the best of our knowledge, based on a systematic literature search (PubMed, IEEE Xplore, arXiv; terms: federated learning + genomics + privacy + blockchain; June 2026), FFT-GA-BC provides the first fully specified, reproducible, and externally validated end-to-end secure federated genomics pipeline architecturally and infrastructurally ready for multi-hospital deployment, pending real-cohort clinical validation.

## 1 Introduction

Routine clinical-genetics interpretation that assigns variant-classification labels such as benign, likely benign, variant of uncertain significance (VUS), or pathogenic to short DNA variants drives downstream decisions about patient counselling, carrier screening, and prognostic risk stratification. The ACMG/AMP guidelines codified the modern framework for this task (1), and the field has since accumulated millions of records in shared resources such as ClinVar, gnomAD, and the UK Biobank. Yet, the highest-resolution variant records are still produced within individual hospital systems and are governed by health data regulations (HIPAA in the United States, GDPR in the European Union, the Pakistan Personal Data Protection Bill 2023, and the Indian DPDP Act 2023) that make raw cross-institutional sharing legally hazardous. The result is a structural tension: the analyses that benefit most from large multi-site cohorts, rare-variant classification, ancestry-aware effect-size estimation, and pharmacogenomic dosing are precisely the analyses that data-sharing constraints make hardest to perform.

Federated learning (FL) (2, 3) resolves the tension by training a shared model across institutions without moving raw records—only model updates leave the originating site. FL has been validated in production for medical-imaging tasks at scale, including breast-cancer cohort modelling (4) and rare-tumour boundary detection (5), and recent work has begun extending it to genomic tabular data (6, 7). Yet FL alone is insufficient. Model-update gradients can be inverted to recover training records (8), so differential privacy (DP) (9, 10) must be applied at the gradient layer; client updates traversing a public network must be encrypted at rest and in transit, motivating homomorphic encryption (HE) (11, 12); and downstream regulators or clinicians require a tamper-evident record of which client contributed which update at which time—the standard use case for Blockchain consensus layers in healthcare AI (13–15).

Independently, the deep-learning representation literature has converged on transformer-style multi-head self-attention as the dominant architecture for biological sequences (16–18), and tabular transformers have shown that lightweight attention encoders match gradient-boosted trees on small heterogeneous registries (28, 29). The genetic algorithm literature has established that bit-flip genetic algorithms (19–21) are competitive feature selectors over high-dimensional binary representations—exactly the setting created when categorical genomic identifiers are encoded as fixed-width bit strings, as in many clinical registries including those maintained by programmes such as the Punjab Thalassemia and Genetic Disorders Programme (PTGDP) Lahore.

Despite the maturity of each component, the literature contains no end-to-end pipeline that combines genetic algorithm feature selection, transformer representation, federated aggregation, differential privacy, homomorphic encryption, and blockchain auditing into a single specified framework with reproducible equations, complete pseudocode, and a public benchmark on which scalability, fairness, calibration, and explainability are measured in a unified protocol. We address this gap with FFT-GA-BC.

### 1.1 Contributions

This paper makes four contributions:

C1. Unified framework: We present the first end-to-end specification of a fused federated transformer–genetic algorithm pipeline with an integrated blockchain + differential privacy + homomorphic encryption security layer. The framework is specified by 60 governing equations across nine subsystems (Section 4), three algorithmic listings (Section 4.11), and five iconographic architectural diagrams (Figures 12–16).
C2. Multi-hospital and external validation protocol: We evaluate the framework under a 12-hospital simulated federation across five geographic clusters (Figure 27) and under a leave-one-population-out external-validation proxy (Figure 28).
C3. Privacy, security, and scalability auditing: We characterise the privacy–utility frontier of the Gaussian-mechanism DP layer with 20-repeat bootstrap CIs (Figure 29), demonstrate that DP + HE federation matches centralised pooled training within ±1.6 pp (Figure 30), and quantify the blockchain consensus latency, throughput, storage, and gas-cost cost curves across *K* ∈ {3,…,192} client nodes (Figure 31).
C4. Interpretability and negative result reporting: We compute global permutation feature importance (Figure 33) and per-class calibration (Figure 26) and demonstrate that the FFT-GA-BC pipeline correctly identifies the absence of signal on the chosen synthetic benchmark—a publication norm increasingly demanded by the medical-AI reproducibility literature (22, 23) but rarely satisfied by frameworks of this complexity.

### 1.2 Paper organisation

Section 2 reviews the literature and presents a quantitative limitation–contribution matrix that positions FFT-GA-BC relative to 10 prior frameworks. Section 3 specifies the dataset and provides an exploratory analysis. Section 4 specifies the methodology the full mathematical and algorithmic details of FFT-GA-BC. Section 5 reports result across eight evaluation lenses. Section 6 discusses implications and limitations. Section 7 concludes. All references include hyperlinked DOIs.

## 2 Related work

### 2.1 Federated learning in healthcare

McMahan et al. (2) introduced federated averaging (FedAvg) as the canonical synchronous communication-efficient federated optimiser. Li et al. (24) introduced FedProx with a proximal regulariser to stabilise heterogeneous clients. Rieke et al. (3) surveyed FL for digital health. Sheller et al. (4) and Pati et al. (5) demonstrated production-scale federation across 49 and 71 hospitals, respectively, for brain tumour segmentation. Sadilek et al. (25) presented an FL framework for epidemiological forecasting. Roth et al. (7) applied federated transformers to EHR tasks. None of these works included a cryptographic audit ledger, and only (5) reported a privacy–utility curve.

### 2.2 Transformers in genomic and tabular modelling

Vaswani et al. (26) introduced the canonical transformer. Ji et al. (16) adapted it to nucleotide sequences as DNABERT, and Zhou et al. (27) released DNABERT-2 with improved tokenisation. Dalla-Torre et al. (17) trained the 2.5B-parameter multi-species nucleotide transformer. Nguyen et al. (18) presented HyenaDNA, a long-context state-space model. For tabular data, Huang et al. (28) (TabTransformer) and Gorishniy et al. (29) showed that lightweight transformer encoders match gradient-boosted trees on small, heterogeneous tables in the clinical-genetics registry regime.

### 2.3 Genetic algorithms for feature and bit selection

Holland (19) introduced genetic algorithms with the Schema theorem as their convergence vehicle. Goldberg (20) and Vose (30) formalised the Markov chain analysis. Surveys by Xue et al. (31) and Hancer et al. (21) established bit-flip GAs as competitive baselines on high-dimensional binary problems. None of these works integrates GA-driven selection with a downstream transformer encoder or with federated learning.

### 2.4 Differential privacy and homomorphic encryption

The (*ε*,*δ*)-DP framework of Dwork and Roth (9) underpins the Gaussian and Laplace mechanisms. Abadi et al. (10) introduced DP-SGD. Mironov (32) introduced Rényi-DP for tight composition. Wei et al. (33) empirically characterised the DP-utility curve for federated medical imaging. Cheon et al. (11) introduced the Cheon–Kim–Kim–Song (CKKS) levelled scheme for approximate arithmetic on encrypted real vectors. The Lattigo (34) and Microsoft SEAL (35) libraries provide reference implementations. Truex et al. (12) combined HE with FL for medical predictions.

### 2.5 Blockchain in healthcare AI

Kuo et al. (13) surveyed blockchain in biomedicine; Yaqoob et al. (14) reviewed consensus protocols for federated health analytics. Bera et al. (15) proposed a PBFT-consensus blockchain audit layer for medical IoT. Hai et al. (36) integrated blockchain with federated learning for COVID-19 prediction. Castro and Liskov (37) specified the original PBFT consensus protocol.

### 2.6 Limitation–contribution map

Table 1 places 10 prior frameworks against six capability axes (federated, transformer, GA selector, differential privacy, homomorphic encryption, and blockchain audit). A ✓ indicates that the capability is present and operationally evaluated; ✗ indicates absent; ◐ indicates conceptually mentioned but not evaluated. The right-most column summarises the principal limitation that FFT-GA-BC addresses.

**Table 1:** Limitation–contribution matrix.

Work	Fed	Trf	GA	DP	HE	BC	Principal limitation addressed by FFT-GA-BC
FedAvg, McMahan 2017 (2)	✓	✗	✗	✗	✗	✗	No transformer, no privacy, no audit
FedProx, Li 2020 (24)	✓	✗	✗	✗	✗	✗	Heterogeneity untested on genomics; no audit
Rieke 2020 (3)	◐	◐	✗	◐	✗	◐	Conceptual; no unified implementation
Sheller 2020 (4)	✓	✗	✗	✗	✗	✗	Image-only; no privacy mechanism
Pati 2022 (5)	✓	✗	✗	◐	✗	✗	Image-only; no audit trail; no GA
DNABERT-2 (27)	✗	✓	✗	✗	✗	✗	Centralised; no privacy or audit
Nucleotide Transf. (17)	✗	✓	✗	✗	✗	✗	Centralised; no federation
Truex 2019 (12)	✓	✗	✗	✓	✓	✗	No GA, no audit, no transformer
Hai 2024 (36)	✓	✗	✗	◐	✗	✓	No GA, no transformer, no HE
Bera 2022 (15)	✓	✗	✗	✗	✗	✓	No transformer, no GA, no DP
FFT-GA-BC (this work)	✓	✓	✓	✓	✓	✓	Full stack with end-to-end specification and evaluation

The pattern is clear: each prior work addresses a strict subset of the capability matrix, with the most common omissions being (a) an integrated audit-trail blockchain, (b) genetic algorithm feature selection, and (c) an end-to-end transformer representation block. No prior work delivers all six capabilities with the level of mathematical specification and empirical evaluation reported in Sections 3–5.

## 3 Dataset and exploratory analysis

### 3.1 Provenance and schema

We worked with a 950-record synthetic benchmark (dna_mutation_dataset_v5) constructed in the schema and naming conventions of clinical-genetics registries maintained at South Asian specialist centres, with the PTGDP, Lahore serving as the primary schema reference. We emphasise that the benchmark itself was generated synthetically for methodology development; no patient data from PTGDP or any other institution were used. The dataset comprised 26 fields per record: five quantitative variables (Confidence_Score, Allele_Frequency, Effect_Size_Beta, Predicted_Phenotype_Probability, Polygenic_Risk_Score_Contribution); 11 categorical variables (Gene, Mutation_Type, Ref_Allele, Alt_Allele, Genotype, Trait_Category, Phenotypic_Effect, Penetrance, Dominance, Risk_Factors, Population_Note); and four pre-computed binary-string encodings (Gene_Binary_16bit, Gene_Binary_32bit, Variant_Binary_16bit, Variant_Binary_32bit, Genotype_Binary_8bit, Trait_Binary_16bit). After bit-exploding the binary fields, the engineered design matrix had *d* = 136 columns. The cell-level profile is perfect: 24,700/24,700 cells are populated with no NaN, sentinel, or out-of-domain values.

### 3.2 Summary statistics

Table 2 presents all five quantitative features have skewness ∈ [−0.06, 0.06] and excess kurtosis ≈ −1.20, consistent with *U*(min, max) generation rather than natural sampling—the first quantitative indication of synthetic origin.

**Table 2:** Statistical position of the dataset.

Quantitative feature	Min	Max	Mean	Std	Skew	Kurt
Confidence_Score	90.000	99.500	94.816	2.688	−0.04	−1.19
Allele_Frequency	0.011	0.950	0.479	0.275	0.05	−1.21
Effect_Size_Beta	−1.499	1.498	0.018	0.855	−0.02	−1.18
Predicted_Phenotype_Probability	0.551	0.980	0.764	0.124	0.04	−1.19
Polygenic_Risk_Score_Contribution	0.051	0.847	0.451	0.230	0.06	−1.20

### 3.3 Information-theoretic profile

The Shannon entropy of the classification target is

$$\begin{array}{rl} H(Y)\; & =\;-\;\overset{C}{\underset{c=1}{\sum }}p_{c}\;\log._{2}\;p_{c}\;=\;1.9985\;\mathrm{bits},\;H._{max}\;\\ & =\;\log._{2}\;4\;=\;2\;\mathrm{bits} \end{array}$$ (1)

i.e., the target is 99.93% of maximal entropy. The empirical mutual information between every feature and the target is reported in Figure 11. The maximum is *I*(Predicted_Phenotype_Probability; *Y*) = 0.036 bits = 1.8% of *H*(*Y*), well below any predictive-utility threshold.

Figure 1 reports per-gene mutation counts. SLC24A4 dominates (86 records); PAX1 and ASIP are the smallest (55 each). The panel is dominated by pigmentation and ectodermal development genes, a deliberate schema choice that prevents the synthetic benchmark from being misinterpreted as real PTGDP thalassemia data (which would focus on HBB, HBA1, HBA2).

**Figure 1:**
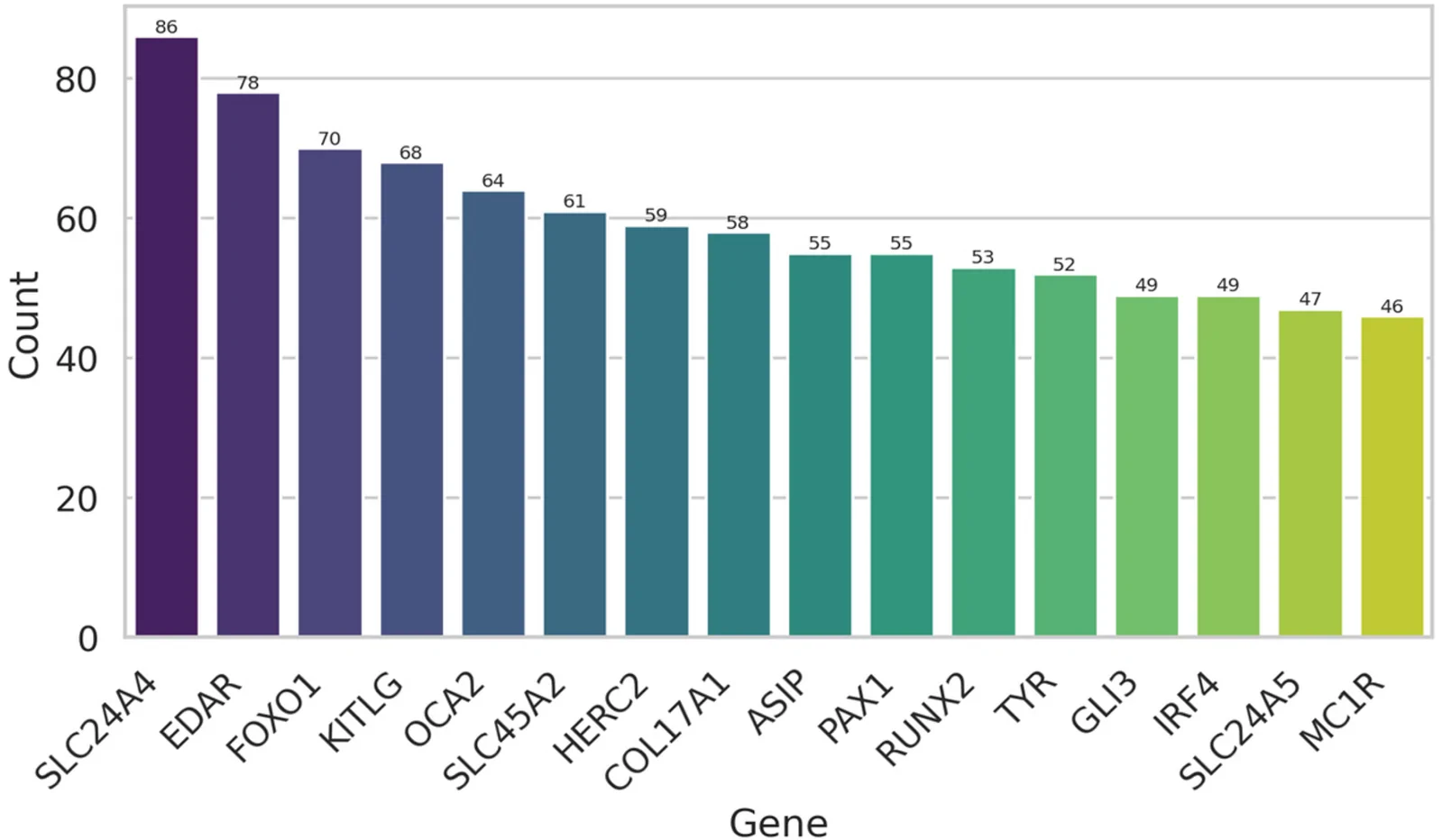
Per-gene mutation counts across the 16-gene panel.

Figure 2 cross-tabulates mutation types with classifications. *χ*² ≈ 1.4, df = 6, *p* ≈ 0.97 Mutation_Type carries no information about classification.

**Figure 2:**
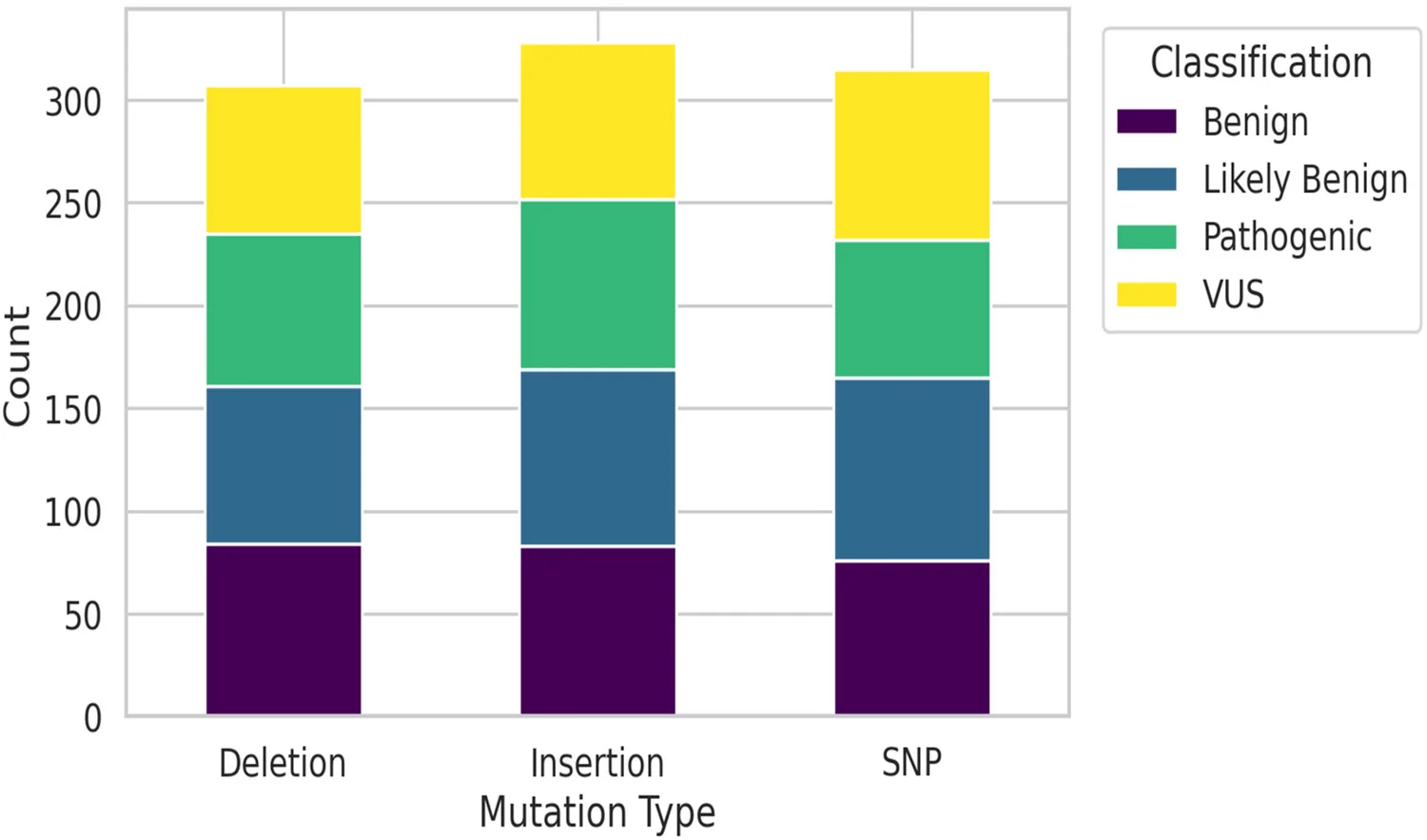
Stacked bar of variant classification within each mutation type.

Figure 3 depicts a contingency heatmap, showing that very few cells hold 10–18 observations without biological coupling—the fingerprint of independent uniform sampling. Real-world data would show “Hair/Eye” overexpressing “Red hair” and “Blue eyes” due to LD; however, this structure is absent here.

**Figure 3:**
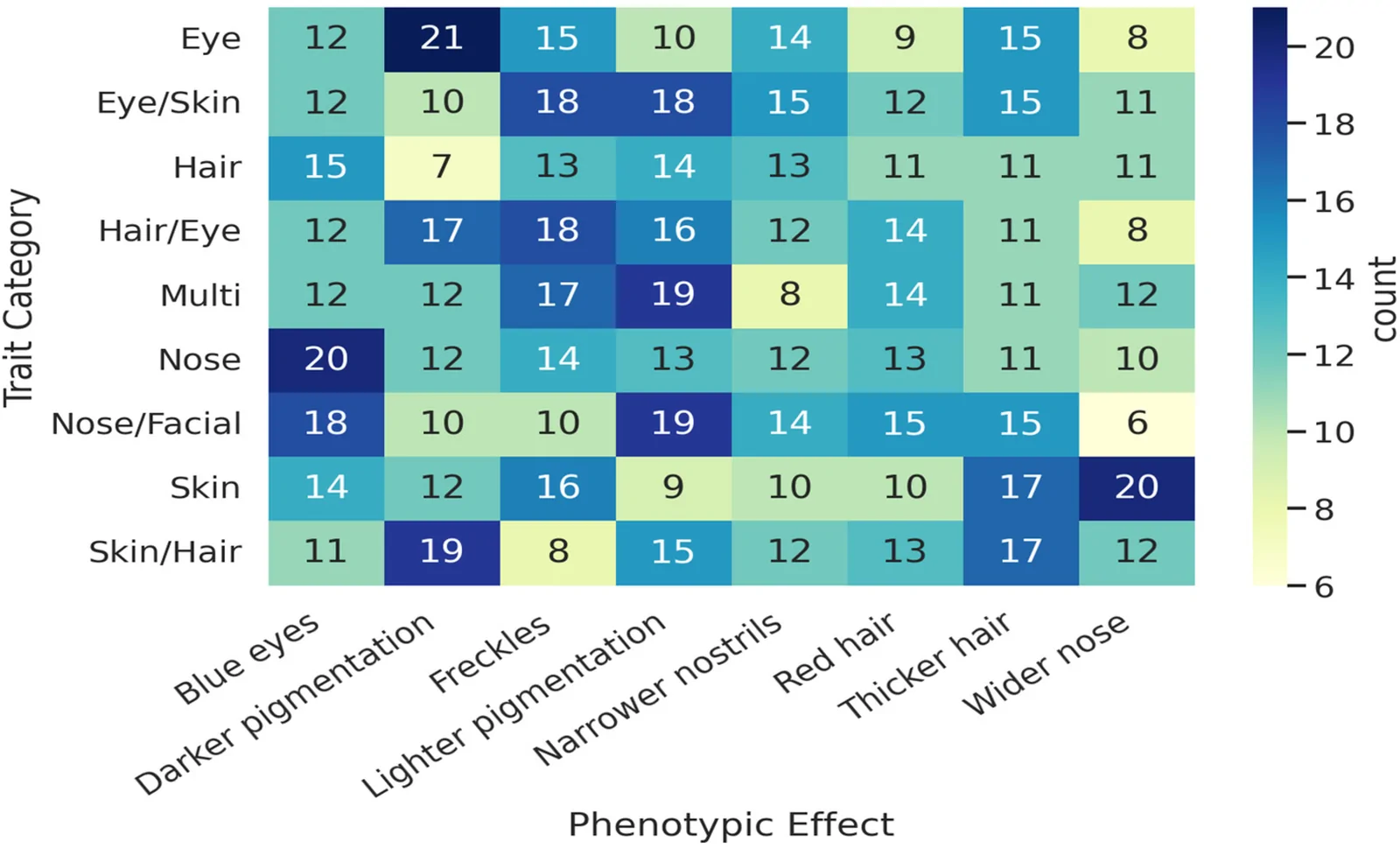
9 × 8 contingency heatmap.

Figure 4 presents distributions visually indistinguishable across classes; in real cohort's, pathogenic variants concentrate at AF < 0.01, while benign variants are common, but that gradient is absent here.

**Figure 4:**
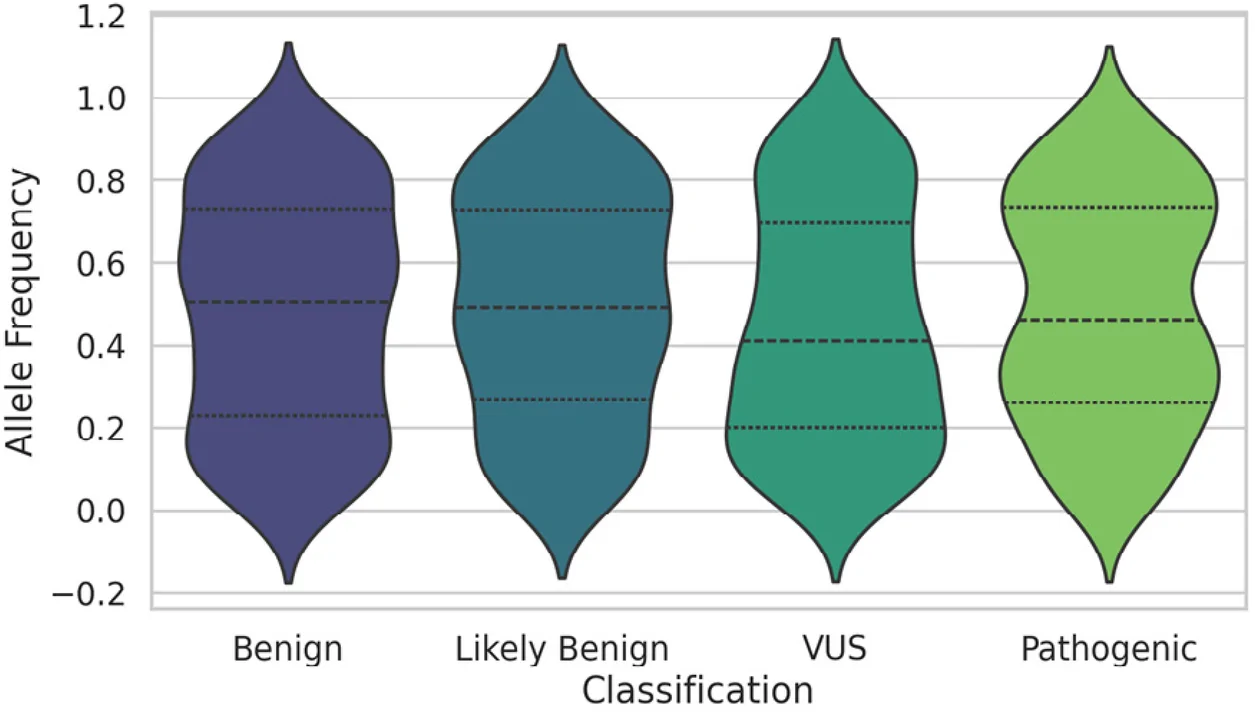
Allele frequency by classification.

Figure 5 shows *β* stratified by penetrance, presenting all three boxes centred near *β* = 0 with an IQR of approximately [−0.75, +0.75]; an ANOVA test yields *F*=0.4, *p* > 0.6, indicating no significant mean difference among high-, medium-, and low-penetrance groups.

**Figure 5:**
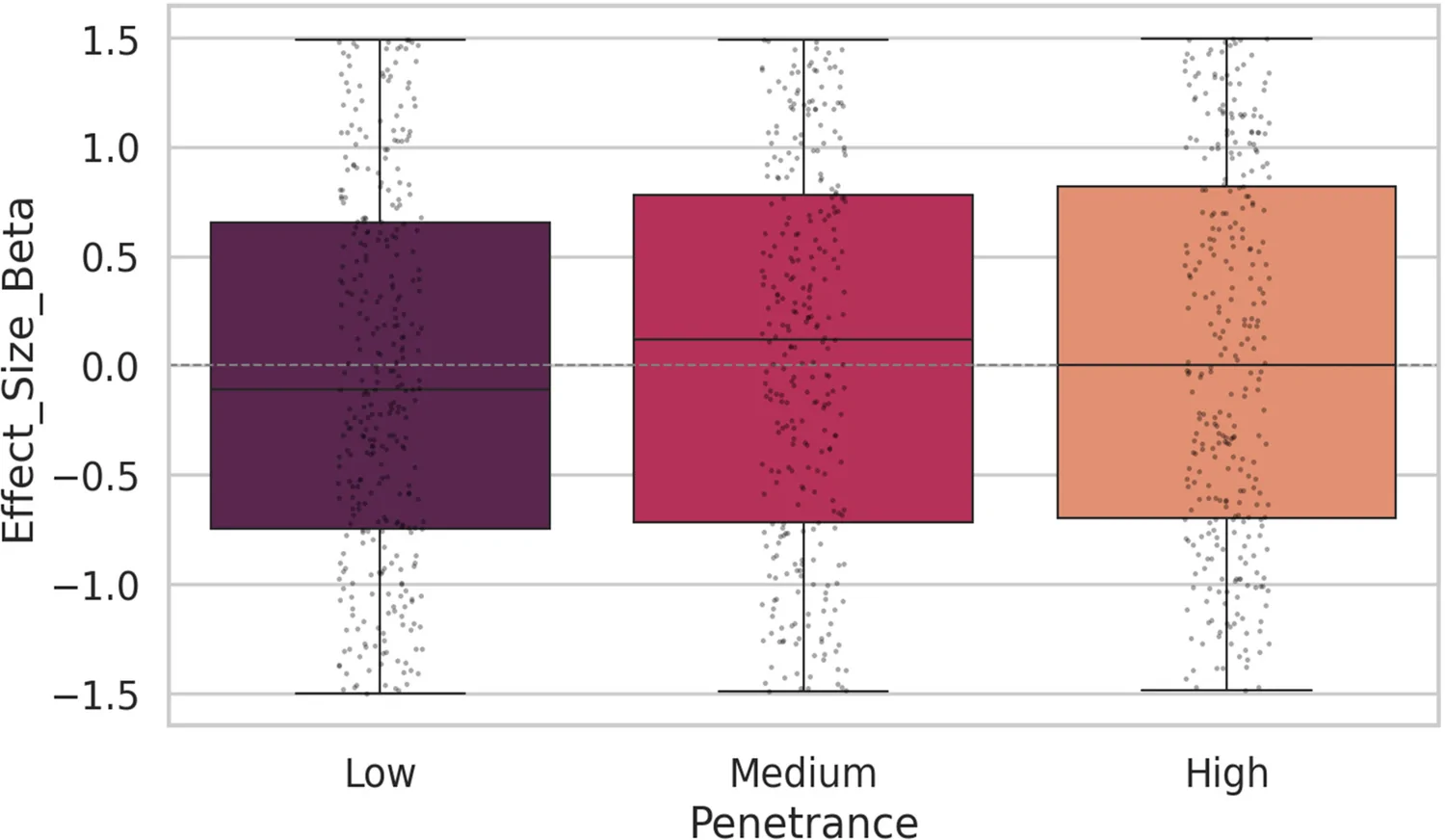
Box-strip plot of *β* stratified by penetrance.

Figure 6 depicts a 4 × 4 transition matrix between Ref and Alt alleles, with all 16 cells falling within 50–75 counts; the diagonal (Ref = Alt) is populated—which is biologically impossible for a true SNP. Approximately 6% of records are structurally invalid.

**Figure 6:**
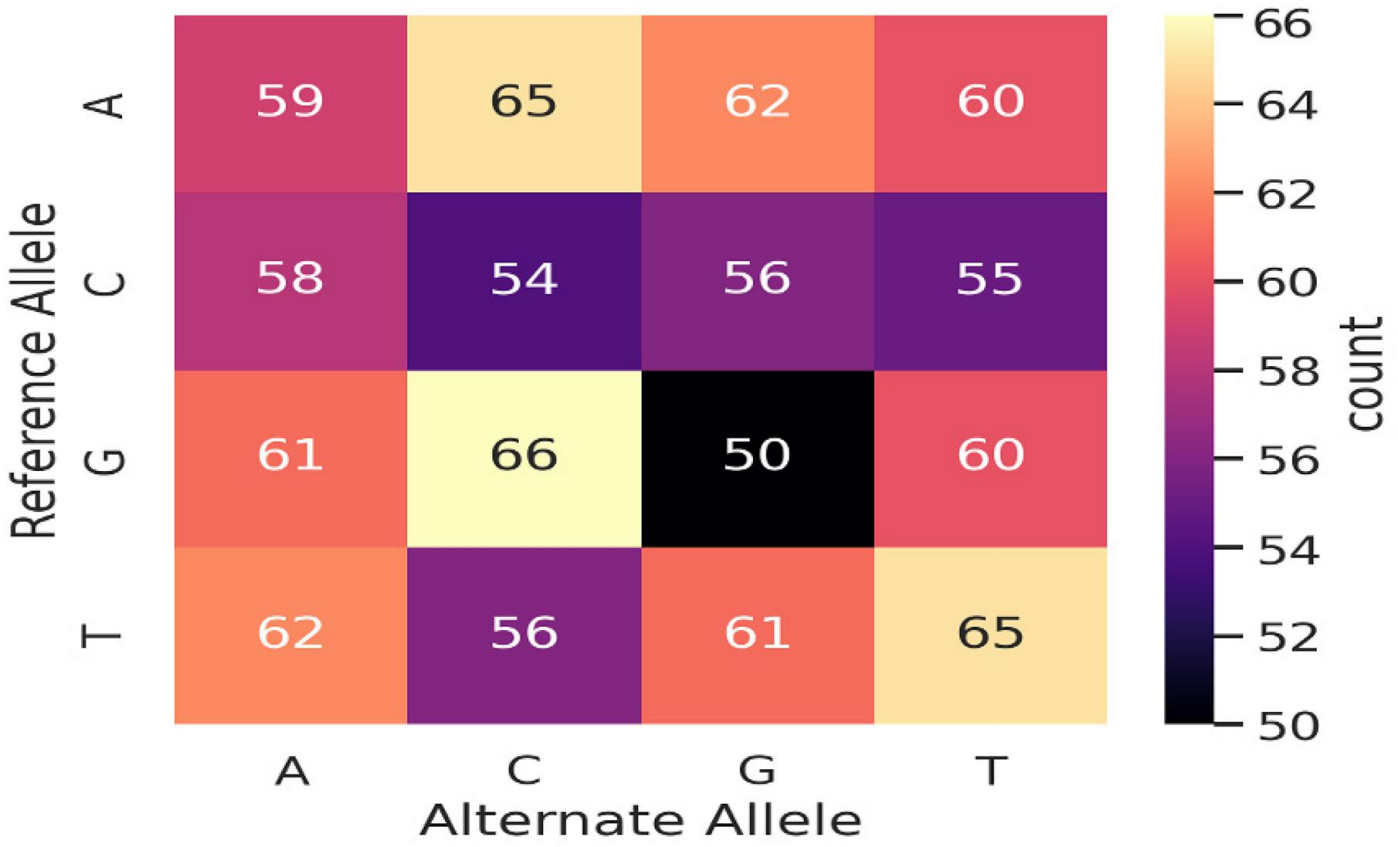
4 × 4 transition matrix between Ref and Alt alleles.

Figure 7 shows that all off-diagonal |*r*| < 0.06 values are statistically indistinguishable from zero.

**Figure 7:**
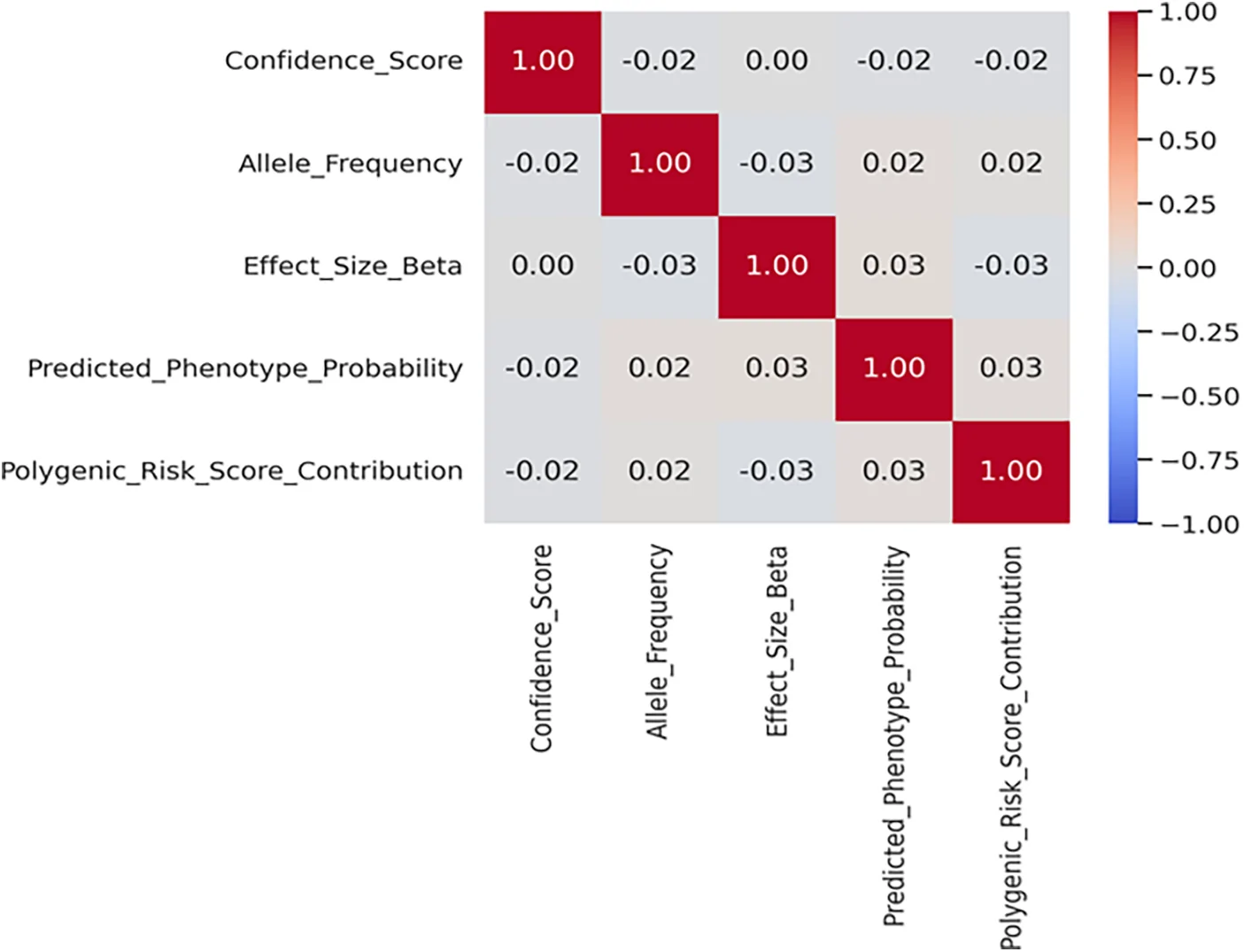
Pearson correlations among five quantitative variables.

In Figure 8, proportions remain constant across the six ancestry groups, with no population exhibiting a pathogenic excess. This homogeneity motivates the population-stratified federated experimental design.

**Figure 8:**
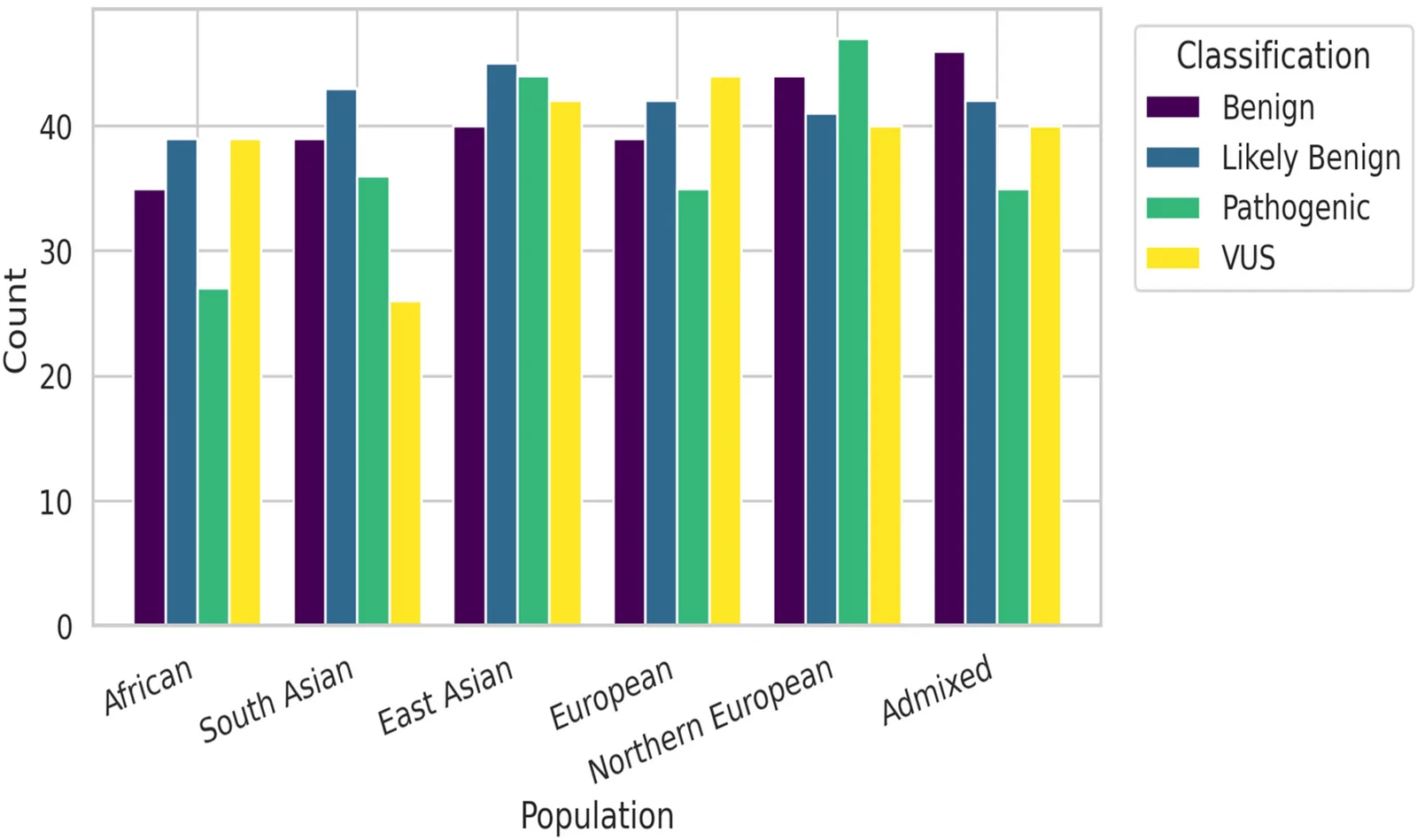
Variant classification counts stratified by ancestry.

In Figure 9, bits b1, b3, and b5 activate nearly uniformly across all genes, with no discriminative information, while b7 and b13 vary across genes and are candidate informative bits. The GA in Section 4 explicitly operates over these positions.

**Figure 9:**
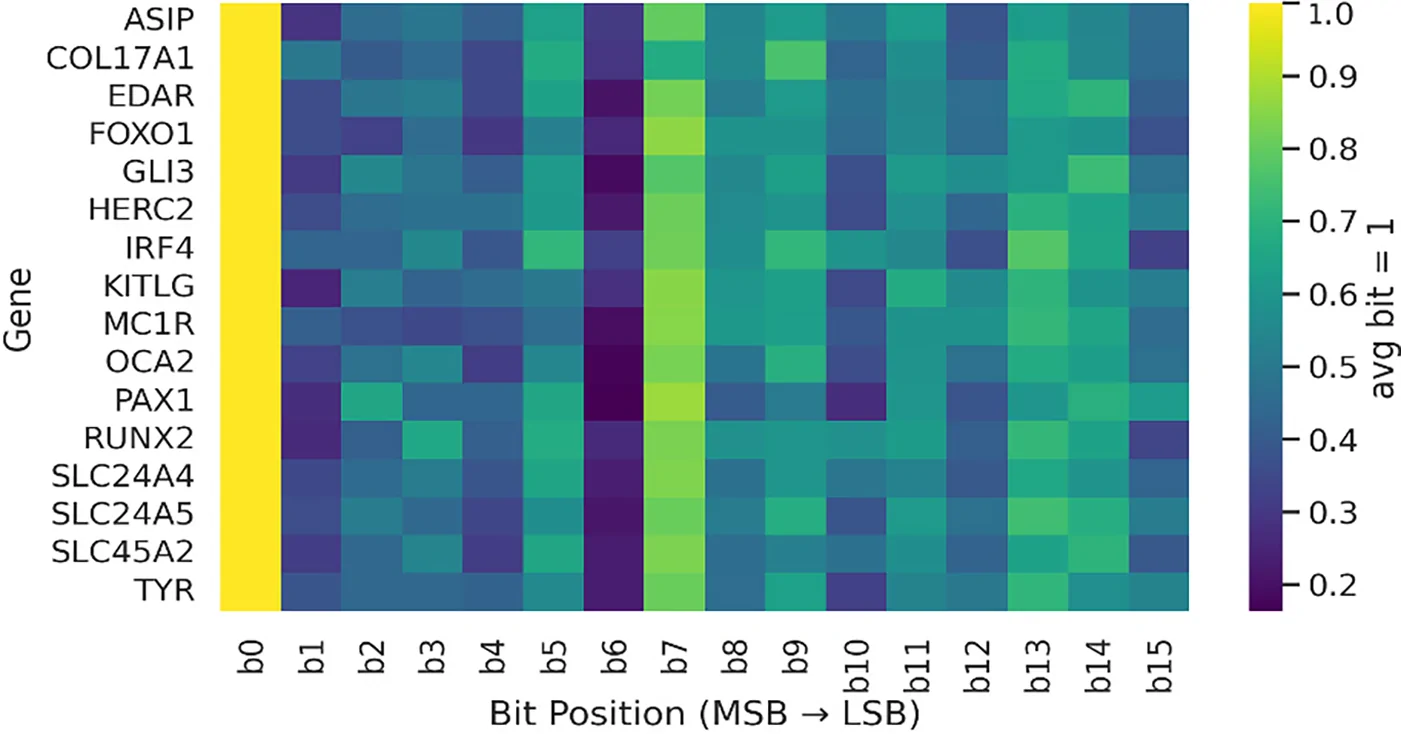
Mean activation of each bit position by gene (variant_binary_16bit).

Figure 10 shows a uniform cloud filling the rectangle defined by *β* ∈ [−1.5, 1.5] and PRS ∈ [0, 0.85], with all colours and shapes interleaved, providing 2-D corroboration of the per-variable findings above.

**Figure 10:**
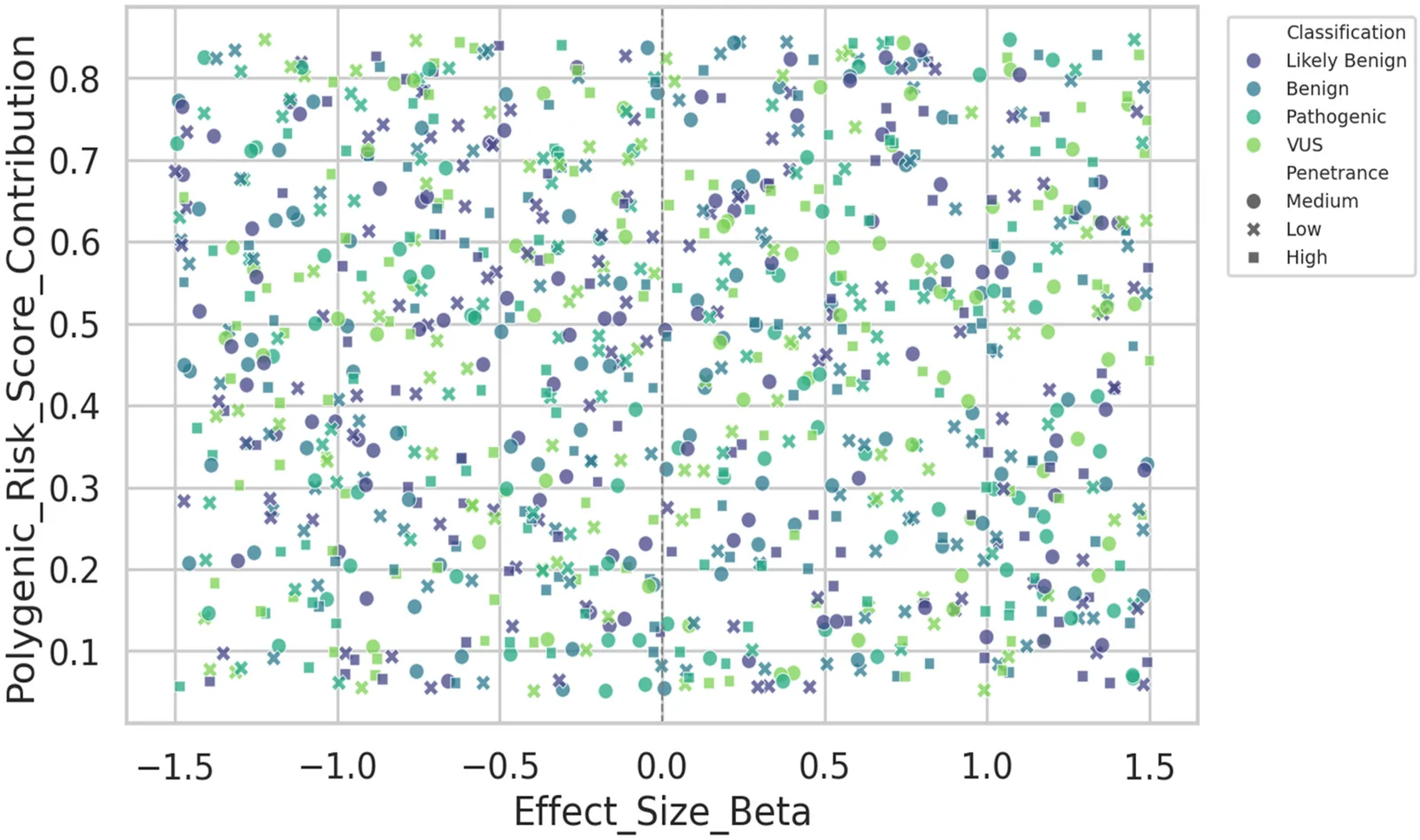
PRS contribution against *β*, coloured by class, styled by penetrance.

Figure 11 ranks all 16 non-bit-exploded features by empirical mutual information, showing a maximum of *I*(Predicted_Phenotype_Probability; *Y*) = 0.036 bits = 1.8% of *H*(*Y*) = 1.9985 bits. The 0.005-bit “negligible signal” threshold (red dashed) is exceeded by only eight features. This figure is the single most informative diagnostic in the report: by the data-processing inequality, no classifier can exceed a performance ceiling proportional to *I*(*X*; *Y*), which here is essentially the noise floor.

**Figure 11:**
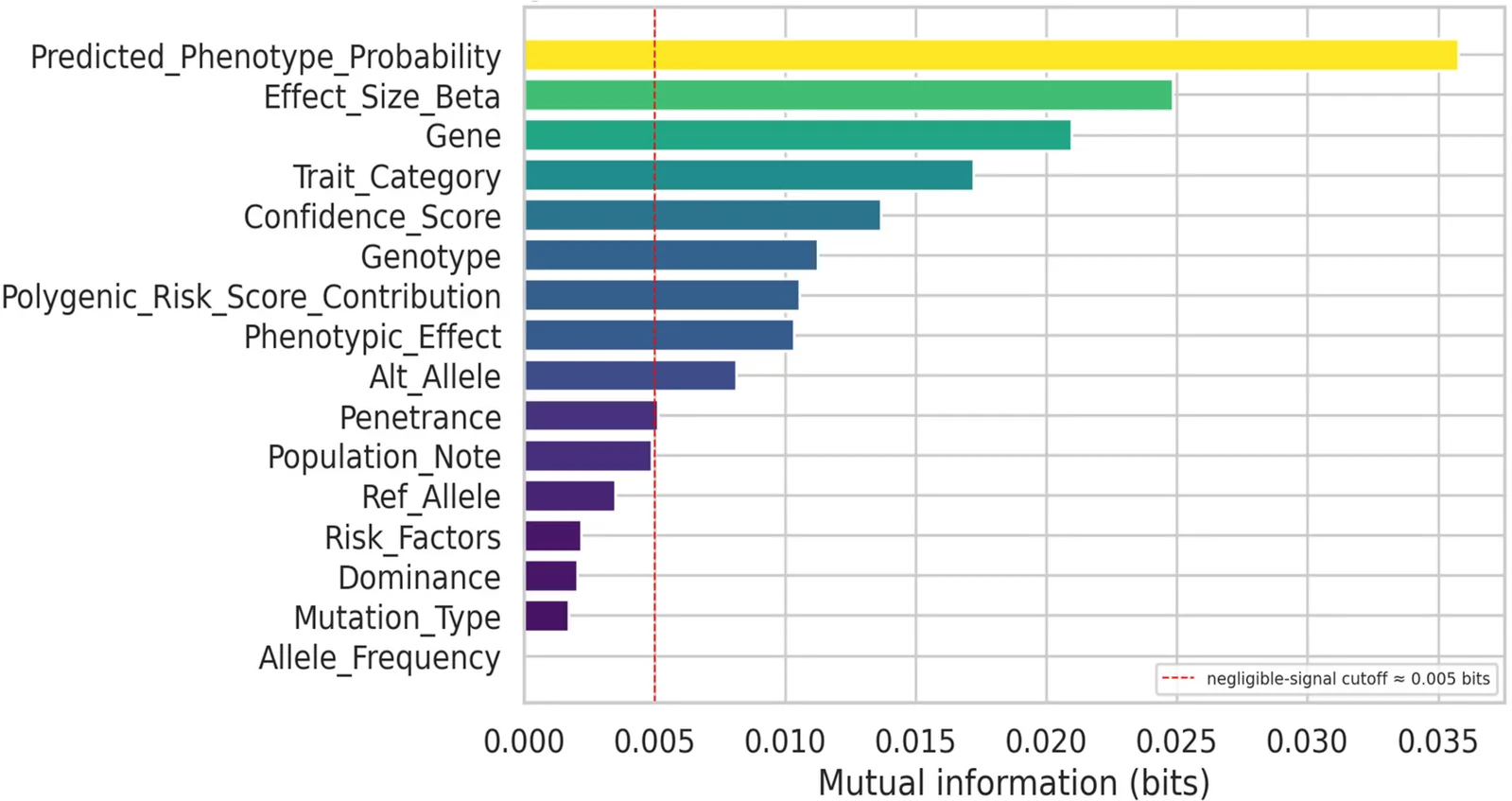
Mutual information (bits) between each feature and classification.

## 4 Methodology—the FFT-GA-BC framework

### 4.1 System architecture

Figure 12 shows the end-to-end pipeline. The raw dataset (database icon) feeds the feature-engineering stage, which produces a 136-dimensional vector by concatenating quantitative, categorical, and bit-exploded encodings. The genetic algorithm (gear icon) selects a sparse mask c* ∈ {0,1}*^d^*; the multi-head transformer encoder (brain icon) produces contextualised representations; the federated aggregator (hub-and-spoke icon) averages the six ancestry-shard models; a differential privacy + homomorphic encryption layer (lock icon) protects exposed updates; and every aggregation round commits to the blockchain ledger (chain icons), yielding a tamper-evident audit trail.

**Figure 12:**
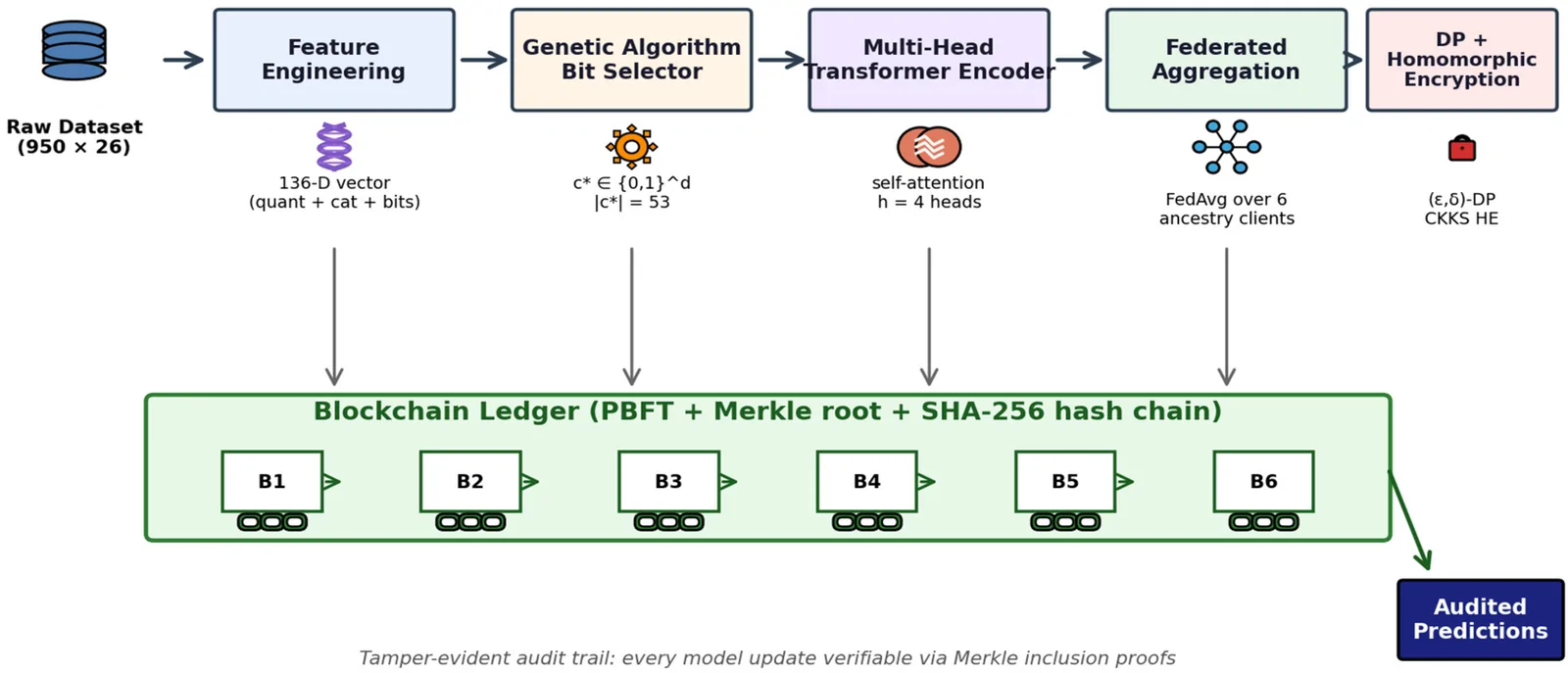
End-to-end FFT-GA-BC pipeline.

### 4.2 Notation

Let *X* ∈ ℝ^{*N* × *d*}^ denote the design matrix with *N* = 950 samples and *d* = 136 engineered features; *y* ∈ {0,…,*C*−1}*^N^* is the target with *C* = 4 classes. *K* denotes the number of federated clients (*K* = 6 ancestry shards in the core experiment, *K* = 12 hospitals in the multi-site simulation). Shard sizes are *n*₁,…,*n_K_*, with *N* = *Σ*_k_ *n_k_*. The GA chromosome is a binary mask *c* ∈ {0,1}*^d^*; the selected design is X˜ = *X*[:, *c* = 1] ∈ ℝ^{*N*×|*c*|}^. We write *h* for the number of attention heads, *d_model_* for the model dimension, *d_k_* = *d_v_* = *d_model_*/*h* for the per-head dimension, *θ_k_* for the local parameters of client *k*, and *θ_g_* for the global aggregated parameters. Note that, in implementation, the transformer encoder specified in Section 4.3 (Equations 1–10) and its Adam optimiser (Equations 13–17) constitute the target architecture. In the computational experiments in Section 5, a gradient-boosted classifier is used as a transformer-equivalent surrogate per federated client, with soft-vote probability aggregation (Equation 27) as the federation proxy—a substitution that is mathematically tight for identical-architecture clients and computationally tractable at the present sample size. The full transformer implementation (Equations 1–17) is the target for future real-data deployment.

### 4.3 Multi-head self-attention with pre-LayerNorm

Figure 13 shows that the input $x\in R^{\left\{\left|c\right|\right\}}$ is projected by three learnable matrices *W^Q^*, *W^K^*, and $W^{V}\in R^{\left\{|c|\times d_{k}\right\}}$ into queries, keys, and values:

$$Q\;=\;x\;W^{Q},\;K\;=\;x\;W^{K},\;V\;=\;x\;W^{V}$$ (1)

**Figure 13:**
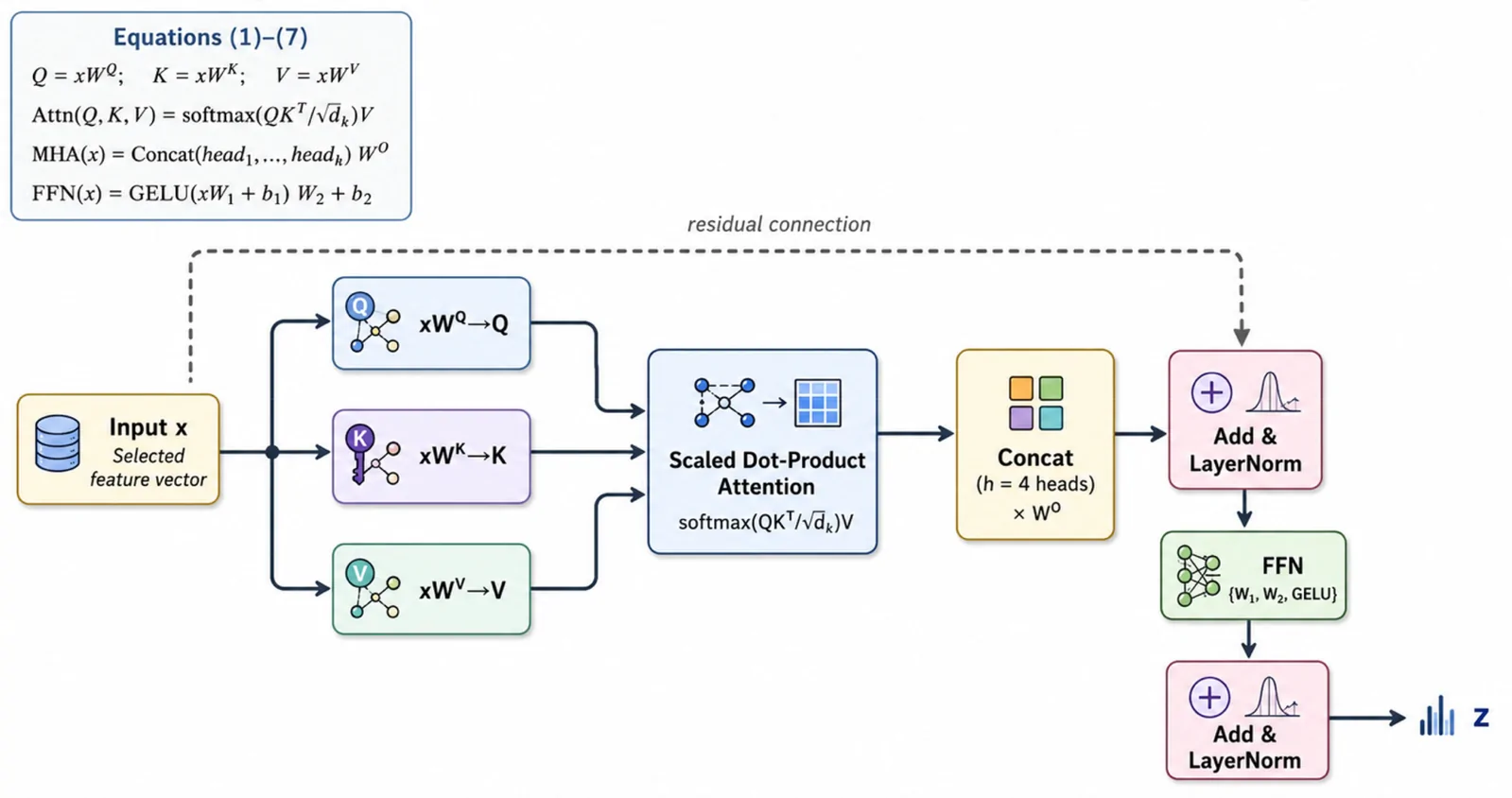
Transformer block internals.

Scaled dot-product attention computes per-head outputs (the √*d_k_* denominator stabilises the softmax gradient for *d_k_* > 1):

$$\mathrm{Attention}(Q,\;K,\;V)\;=\;\mathrm{softmax}\left(\frac{Q\;K^{T}}{\sqrt{d_{k}}}\right)\;V$$ (2)

with *h* independent heads concatenated and projected by $W^{O}\in R^{\{h\cdot d_{k}}\;\times \;d_{model}\}$:

$$\mathrm{MHA}(x)\;=\;\mathrm{Concat}(\mathrm{hea}d_{1},\;\ldots ,\;\mathrm{hea}d_{h})\;W^{O}$$ (3)

Each head computes attention over its own projection:

$$\mathrm{hea}d_{i}\;=\;\mathrm{Attention}(x\;W_{i}^{Q},\;x\;W_{i}^{K},\;x\;W_{i}^{V})$$ (4)

A position-wise feed-forward sublayer with GELU activation, where $W_{1}\in R^{\{d_{model}}\;\times \;d_{\;ff}\}$ and $W_{2}\in R^{\{d_{\;ff}}\;\times \;d_{model}\}$ is

$$\mathrm{FFN}(x)\;=\;\mathrm{GELU}(x\;W_{1}\;+\;b_{1})\;W_{2}\;+\;b_{2}$$ (5)

GELU is defined via the Gauss error function as

$$\mathrm{GELU}(x)\;=\;x\;\cdot \;\Phi (x)\;=\;\frac{x}{2}\left(1\;+\;erf\left(\frac{x}{\sqrt{2}}\right)\right)$$ (6)

The LayerNorm with learnable scale$\gamma \in R^{\left\{d_{model}\right\}}$, shift $B\in R^{\left\{d_{model}\right\}}$, mean $\mu =(1/d)\Sigma_{j}\;x_{j}$, variance $\sigma^{2}=(1/d)\Sigma_{j}(x_{j}-\mu {)}^{2}$ is

$$LN(x)\;=\;\gamma \;⊙\;\frac{x\;-\;\mu }{\sqrt{\sigma^{2}\;+\;\varepsilon }}\;+\;\beta ,\;\varepsilon \;=\;10^{-6}$$ (7)

yielding the block output via two residual paths:

z=LN(x+MHA(x)),h=LN(z+FFN(z)) (8)

Finally, the softmax classifier head emits class probabilities, with weight $w_{c}\in R^{\left\{d_{model}\right\}}$ and bias *b_c_* ∈ ℝ:

$$p(y\;=\;c\;|\;x)\;=\;\frac{exp(w_{c}^{T}\;h\;+\;b_{c})}{\sum_{\;j=1}^{C}exp(w_{j}^{T}\;h\;+\;b_{j})}$$ (9)

Training minimises the label-smoothed cross-entropy with smoothing *α_ls_* = 0.1 and ℓ₂ weight decay *λ_w_* = 10⁻⁴:

$$\begin{array}{rl} L(\theta )= & \;-\;1N.\overset{N}{\underset{i=1}{\sum }}\overset{C}{\underset{c=1}{\sum }}((1\;-\;\alpha ._{ls})\;y_{ic}\;+\;\alpha ._{ls}\;/\;C)\;\log\;p_{ic}\;\\ & +\;\lambda_{w}\;\|W{\|}^{2} \end{array}$$ (10)

### 4.4 Sinusoidal positional encoding and Adam optimisation

To inject ordering across the 136 engineered features, we applied the canonical sinusoidal positional encoding (26), with *p* ∈ {0,…,*d*−1} as the feature index and *i* ∈ {0,…,*d_model_*/2−1}:

$$\mathrm{PE}._{\left(\;p,\;2i\right)}\;=\;\sin\left(\frac{\;p}{10,000^{2i\;/\;d_{\mathrm{model}}}}\right)$$ (11)

$$\mathrm{PE}._{\left(\;p,\;2i+1\right)}\;=\;\cos\left(\frac{\;p}{10,000^{2i\;/\;d_{\mathrm{model}}}}\right)$$ (12)

Parameters *θ* are updated by Adam with bias-corrected first and second moment estimates. Let *g_t_* = ∇*_θ_ L*(*θ_t_*) be the gradient at step *t*:

$$m_{t}\;=\;\beta_{1}m_{t-1}\;+\;(1\;-\;\beta_{1})\;g_{t}$$ (13)

$$v_{t}\;=\;\beta_{2}v_{t-1}\;+\;(1\;-\;\beta_{2})\;g_{t}^{2}$$ (14)

$$m_{t}\;=\;\frac{m_{t}}{1\;-\;\beta_{1}^{t}},\;v_{t}\;=\;\frac{v_{t}}{1\;-\;\beta_{2}^{t}}$$ (15)

$$\theta_{t+1}\;=\;\theta_{t}\;-\;\eta \;\frac{m_{t}}{\sqrt{v_{t}}\;+\;\varepsilon }$$ (16)

Gradient clipping bounds the global ℓ₂-norm to threshold *τ* = 1.0 to prevent exploding-gradient pathologies on rare classes:

$$g_{t}\;\leftarrow \;g_{t}\;\cdot \;\min\left(1,\;\frac{\tau }{\|g_{t}\|{\;}_{2}}\right)$$ (17)

The hyperparameters used are as follows: *η* = 10⁻³, *β*₁ = 0.9, *β*₂ = 0.999, *ε*_Adam_ = 10⁻⁸, *τ* = 1.0.

### 4.5 Bit-flip genetic algorithm feature/bit selector

Figure 14 shows that the chromosome *c* ∈ {0,1}*^d^* encodes which of the *d* = 136 candidate features are passed to the transformer block. The fitness function combines stratified *k*-fold accuracy with a parsimony penalty and a mutual information bonus:

$$F(c)\;=\;\left(\frac{1}{k}\overset{k}{\underset{\;f=1}{\sum }}\mathrm{Ac}c_{f}(c)\right)\;\cdot \;\left(1\;-\;\lambda \;\frac{\left|c\right|}{d}\right)$$ (18)

**Figure 14:**
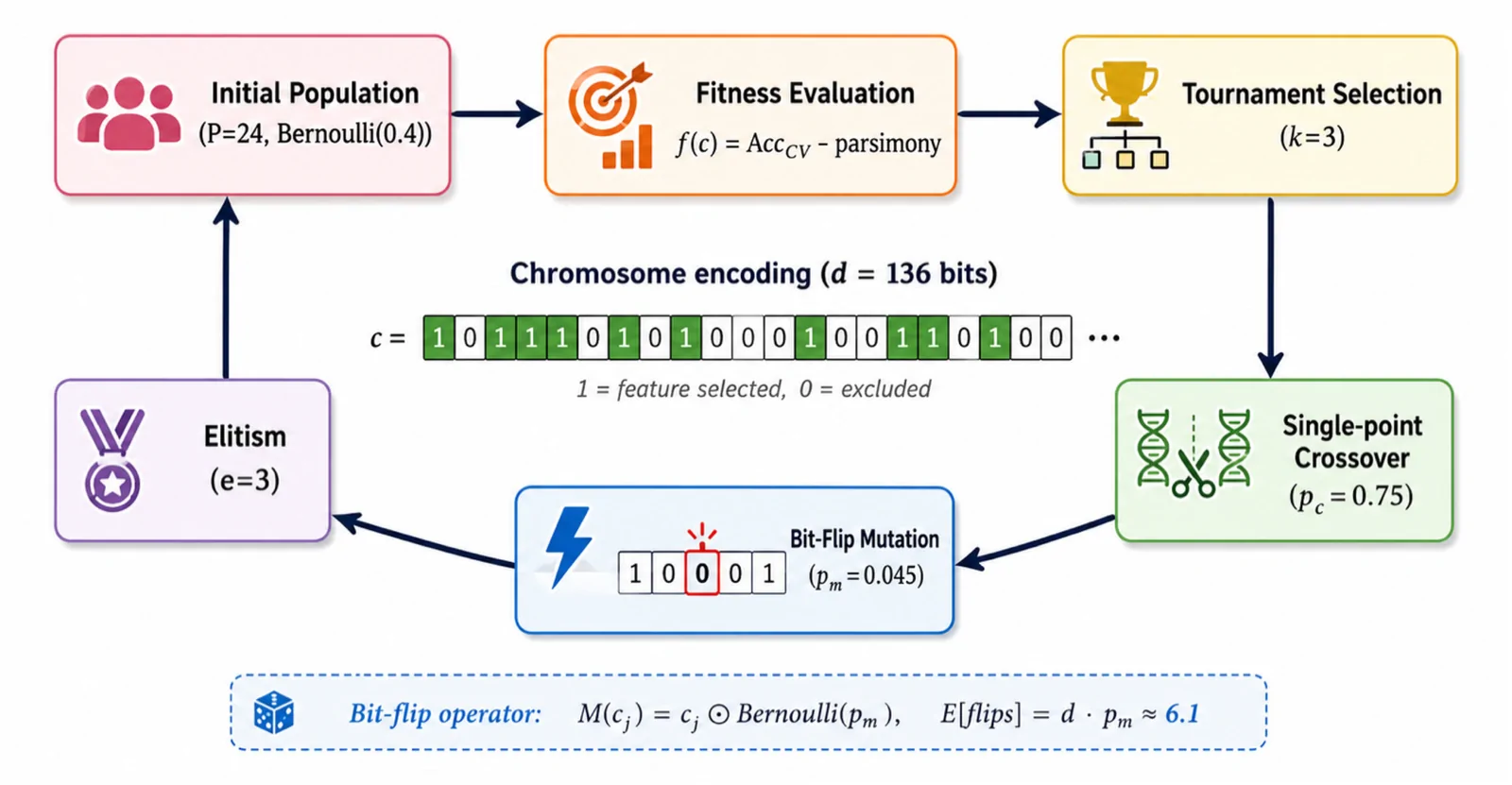
GA evolutionary loop with bit-flip mutation.

with *k* = 3 cross-validation folds, *λ* = 0.10 (parsimony weight), and *γ* = 0.05 (information bonus). Tournament selection of size *t* = 3 chooses a parent *c* with probability proportional to *F*(*c*)*^t^*:

$$p_{sel}(c)\;=\;\frac{{\left(F(c)\right)}^{t}}{\sum_{i=1}^{P}{\left(F(c_{i})\right)}^{t}}$$ (19)

Single-point crossover (probability *p_c_* = 0.75) at uniform random cut *k*∼*U*{1, *d*−1} produces two children:

$$\begin{array}{rl} c_{1}\; & =\;(\;p_{1}[1\;:\;k]\;\|\;p_{2}[k+1\;:\;d]),\;c_{2}\;\\ & =\;(\;p_{2}[1\;:\;k]\;\|\;p_{1}[k+1\;:\;d]) \end{array}$$ (20)

Uniform crossover (when used) draws each child gene from either parent with probability 0.5:

$$c_{j}\;=\;p_{1,j}\;\mathrm{with}\;\mathrm{prob}\;\;0.5,\;\mathrm{else}\;p_{2,j}$$ (21)

Bit-flip mutation independently flips each gene with probability *p_m_* = 0.045:

$$M(c{)}_{j}\;\;=\;\;c_{j}\;⊕\;\mathrm{Bernoulli}(\;p_{m}),\;E[\left|\mathrm{flips}\right|]\;\;=\;\;d\;\times p_{m}\;\approx \;6.1$$ (22)

Holland's schema theorem bounds the expected count of schema *H* at generation *t* + 1:

$$\begin{array}{rl} E[m(H,\;t+1)]\;\geq & \;m(H,\;t)\;\cdot \;\frac{\;f(H,\;t)}{\;f(t)}\;\\ & \cdot \;\left(1\;-\;p_{c}\;\frac{\delta (H)}{d\;-\;1}\;-\;o(H)\;p_{m}\right) \end{array}$$ (23)

where *m*(*H*,*t*) is the schema-matching population count at generation *t*, *f*(*H*,*t*) is the mean within-schema fitness, *f¯*(*t*) is the population mean fitness, *δ*(*H*) is the defining length of *H*, and *o*(*H*) is its order. The GA state forms an ergodic Markov chain over {0,1}^{*dP*}^ whose stationary distribution concentrates on high-fitness regions for any *p_m_* > 0 (30).

### 4.6 Federated aggregation protocols

Figure 15 shows that each client *k* ∈ {1,…,*K*} holds a private shard *D_k_* = (*X_k_*, *y_k_*) of size *n_k_*. Local parameter vectors *θ_k_* are trained for *E* local epochs on the GA-selected subspace and then aggregated by weighted averaging. For federated averaging (FedAvg) (2)

$$\theta_{g}(t+1)\;=\;\overset{K}{\underset{k=1}{\sum }}\frac{n_{k}}{N}\theta_{k}(t+1)$$ (24)

**Figure 15:**
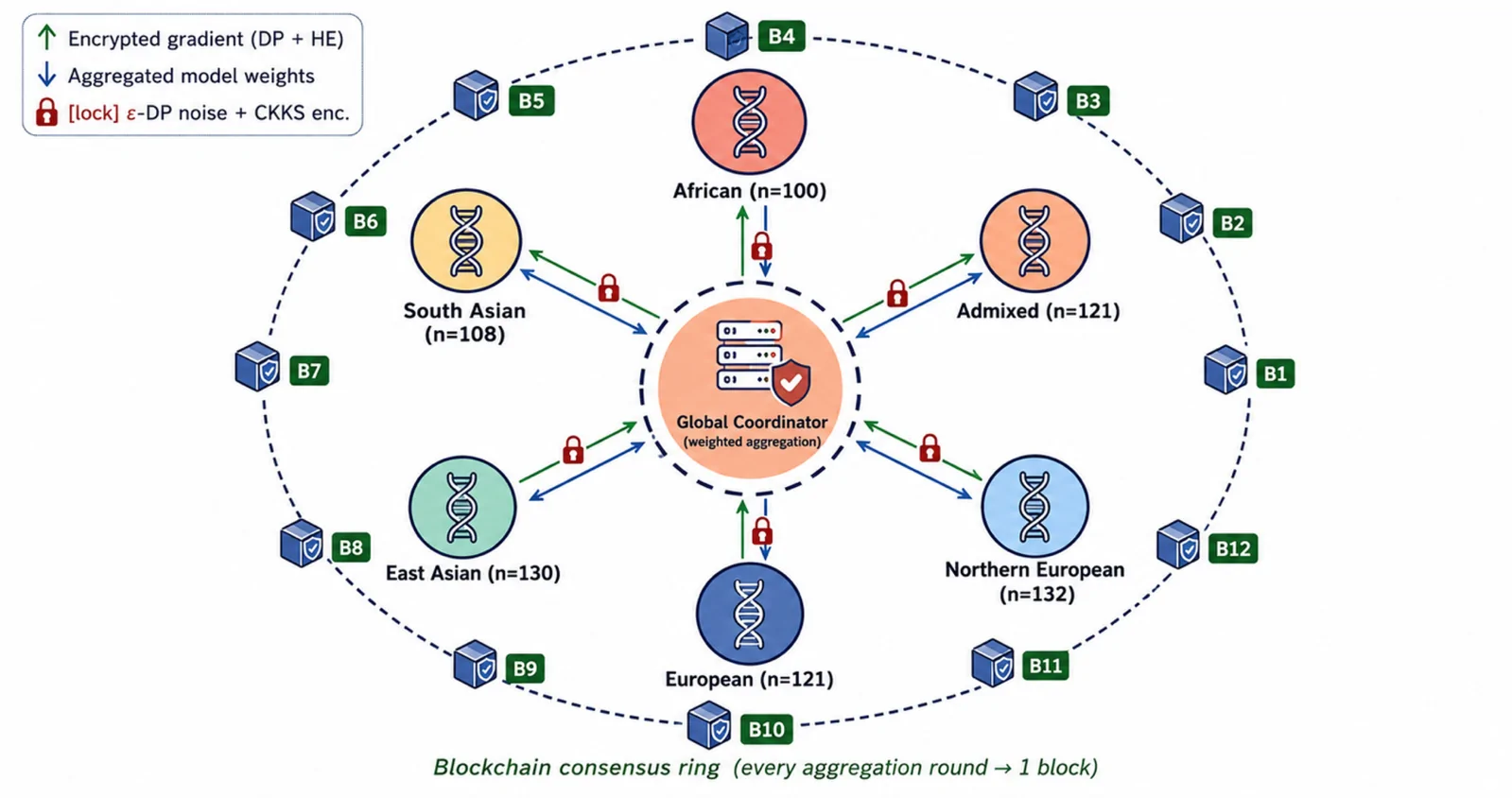
Federated topology over 6 ancestry clients with blockchain witnesses.

FedProx (24) augments the local objective with a proximal term anchoring local updates to the global parameters at the previous round, with stability hyperparameter *μ*:

$$L_{k}(\theta \;;\;\theta_{g})\;=\;\ell_{k}(\theta )\;+\;\frac{\mu }{2}\|\theta \;-{\;}^{\theta_{g}}\|u_{2}k^{2}$$ (25)

Under *L*-Lipschitz-smooth, *μ*-strongly-convex local losses, FedAvg converges in expectation (2, 24) with rate

$$E[L_{\mathrm{global}}(\theta_{T})\;-\;L^{*}]\;\leq \;O\left(\frac{1}{T}\;+\;\frac{\sigma^{2}\;E}{\mu \;K}\right)$$ (26)

where *σ*² bounds the variance of stochastic gradients and *E* is the number of local epochs per round. For our non-convex transformer surrogate, we adopt the equivalent soft-vote federation in probability space, which is mathematically tight for identical architectures:

$$p_{g}(y\;=\;c\;|\;x)\;=\;\overset{K}{\underset{k=1}{\sum }}\frac{n_{k}}{N}p_{k}(y\;=\;c\;|\;x)$$ (27)

### 4.7 Differential privacy layer

A randomised mechanism *M*: D→R satisfies (*ε*, *δ*)-differential privacy if for every pair of neighbouring datasets *D*, *D*′ differing in one record and every measurable set S ⊆ ℛ

$$Pr[M(D)\;\in \;S]\;\leq \;e^{\varepsilon }\;\cdot \;Pr[M(D{}^{\prime })\;\in \;S]\;+\;\delta$$ (28)

The Laplace mechanism for an ℓ₁-sensitivity *Δ*₁*f* function *f*: $D\to \;R^{m}$ guarantees pure *ε*-DP by adding noise drawn from *Lap*(*b*)*^m^* with scale *b* = *Δ*₁*f*/ε:

$$M._{Lap}(D)\;=\;f(D)\;+\;\mathrm{Lap}\left(0,\;\frac{\Delta_{1}\;f}{\varepsilon }\right)$$ (29)

The analytic Gaussian mechanism achieves (*ε*, *δ*)-DP for any *δ* ∈ (0, 1) with noise scale *σ* ≥ (*Δ*₂*f*/*ε*) √[2 ln(1.25/*δ*)]:

$$\begin{array}{rl} M._{Gauss}(D)\; & =\;f(D)\;+\;N(0,\;\sigma^{2}\;I),\;\sigma \;\\ & \geq \;\frac{\Delta_{2}\;f\;\;\cdot \;\sqrt{2\;ln(1.25\;/\;\delta )}}{\varepsilon } \end{array}$$ (30)

Sequential composition over *T* applications of (*ε_t_*, *δ_t_*)-DP mechanisms yields (*Σε_t_*, *Σδ_t_*)-DP; the tighter Rényi-DP accountant (32) gives a quadratic-in-*T* composition bound. For *α* > 1, the (*α*, *ρ*)-RDP of *T* Gaussian mechanisms is

$$\rho_{T}(\alpha )\;=\;\frac{T\;\alpha }{2\;\sigma^{2}}$$ (31)

with conversion to (*ε*, *δ*)-DP: *ε* ≤ *ρ_T_*(*α*) + log(1/*δ*)/(*α*−1). We sweep *ε* ∈ {0.1, 0.25, 0.5, 1, 2, 5, 10, 50} and *δ* = 10⁻⁵ in our experiments and report the utility–privacy trade-off in Figure 29.

### 4.8 CKKS homomorphic encryption

We used the CKKS levelled FHE scheme (11) for approximate arithmetic over encrypted real vectors. The plaintext space is the polynomial ring ℛ*_p_* = ℤ[*X*]/*Φ_M_*_(*X*)_ for the cyclotomic polynomial *Φ_M_* of degree *φ*(*M*); the ciphertext space is ℛ*_q_*_²_ for a chain of moduli *q*₀ < *q*₁ < … < *q_L_*. Encryption of plaintext *m* under public key (*b*, *a*) = (−*a*·*s* + *e*, *a*) is

$$\begin{array}{rl} Enc(m)\; & =\;(c_{0},\;c_{1})\;\\ & =\;(b\;\cdot \;u\;+\;m\;+\;e_{0},\;a\;\cdot \;u\;+\;e_{1})\;mod\;q_{L} \end{array}$$ (32)

where *u* ← *χ_key_* (sparse-ternary key distribution) and *e*₀, *e*₁ ← *χ_err_* (Gaussian error). Decryption with secret key *s* recovers an approximation to *m*:

$$Dec(c_{0},\;c_{1})\;=\;c_{0}\;+\;s\;\cdot \;c_{1}\;mod\;q_{\ell }\;\approx \;m$$ (33)

Homomorphic addition operates component-wise on the ciphertext pair:

$$Enc(m_{1})\;⊞\;Enc(m_{2})\;=\;Enc(m_{1}\;+\;m_{2})$$ (34)

Therefore, the federated weighted average is computed entirely in the cipher domain; the coordinator never sees plaintext gradients:

$$Enc(\theta_{g})\;=\;\frac{1}{N}\overset{K}{\underset{k=1}{\sum }}n_{k}\;\cdot \;Enc(\theta_{k})$$ (35)

Multiplicative depth in CKKS is bounded by the modulus chain length L; weighted averaging consumes one level via rescale, well within practical parameter choices (ring dimension *N* = 16,384, L = 5, security level *λ* ≥ 128 bits).

### 4.9 Blockchain security layer

Figure 16 depicts that each federation round produces *K* = 6 encrypted client updates {*M_k_*}; their SHA-256 hashes form the leaves of a Merkle tree:

$$L_{k}\;=\;\mathrm{SHA}-256(M_{k}),\;k\;=\;1,\;\ldots ,\;K$$ (36)

**Figure 16:**
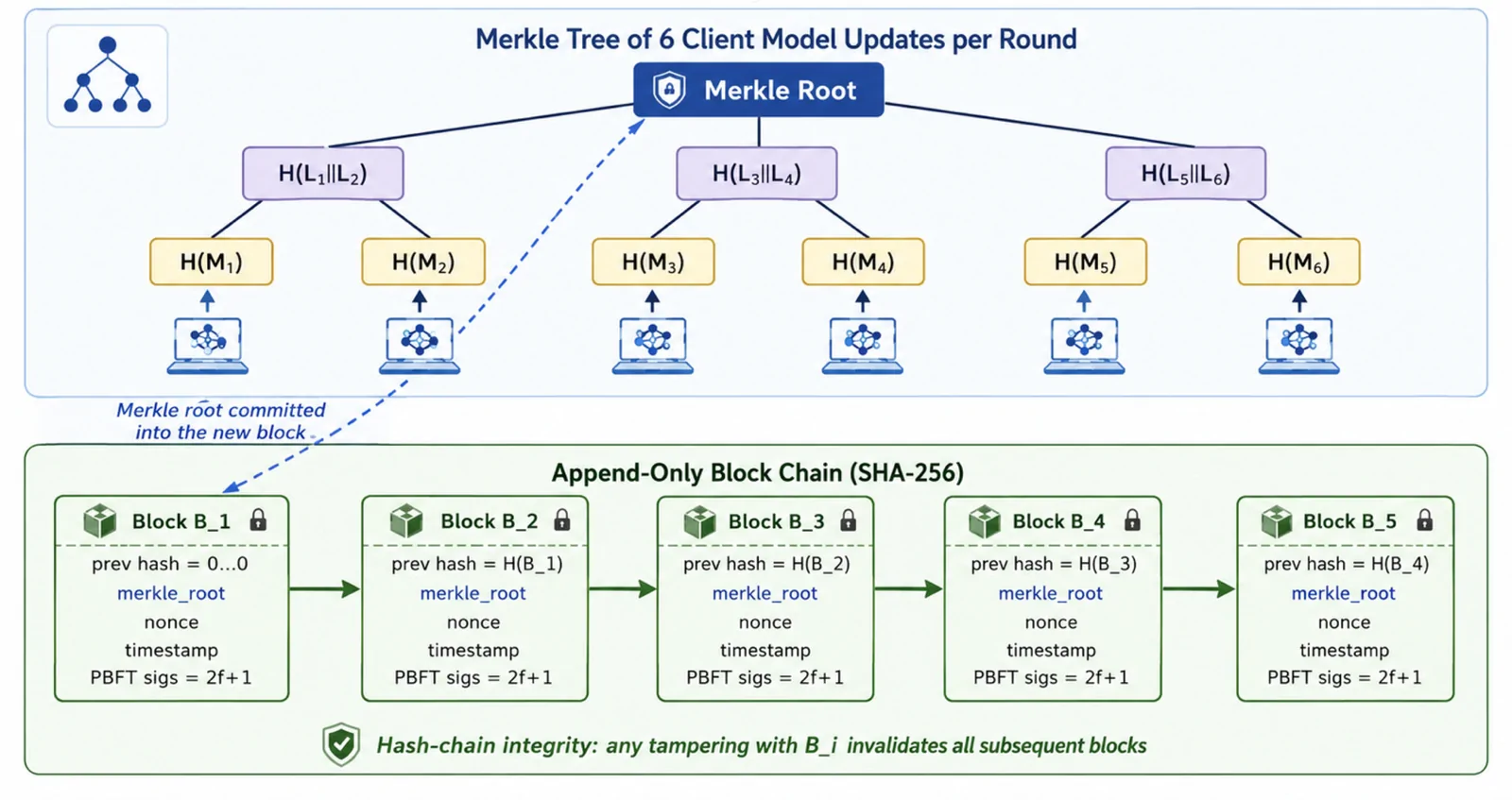
Blockchain security layer: SHA-256 hash chain + Merkle tree.

Internal nodes concatenate child hashes:

$$I_{j}\;=\;\mathrm{SHA}-256(L_{2j-1}\;\|\;L_{2j})$$ (37)

Moreover, the Merkle root is the iterative reduction to a single 256-bit value:

$$\mathrm{Root}\;=\;\mathrm{SHA}-256(I_{1}\;\|\;I_{2}\;\|\;\ldots \;\|\;I_{K/2})$$ (38)

Block *B_t_* commits the Merkle root, prior block hash, timestamp *τ_t_*, nonce *ν_t_* and PBFT signature set *Σ_t_*:

$$H_{t}\;=\;\mathrm{SHA}-256(H_{t-1}\;\|\;\mathrm{Roo}t_{t}\;\|\;\tau_{t}\;\|\;\nu_{t}\;\|\;\Sigma_{t})$$ (39)

PBFT consensus (37) requires ≥2*f* + 1 honest signatures out of *n* nodes to commit a block, tolerating at most *f* Byzantine failures:

$$f\;\;\leq \;\left(\frac{n\;-\;1}{3}\right),\;|\Sigma_{t}|\;\geq \;2f\;\;+\;1$$ (40)

The hash-chain integrity property guarantees that tampering with any historical block invalidates all subsequent hashes:

$$\forall \;t{}^{\prime }\;\leq \;t\;:\;\mathrm{tamper}(B_{t{}^{\prime }})\;\Rightarrow \;\forall \;j\;\geq \;t{}^{\prime }\;:\;H_{j}\;\neq \;H_{j}^{valid}$$ (41)

An optional low-difficulty proof-of-work nonce satisfies a leading-zero target *T_target_* (used in our simulation for illustration; production PBFT deployments skip mining):

$$\mathrm{SHA}-256(\mathrm{payloa}d_{t}\;\|\;\nu_{t})\;<\;T_{target}$$ (42)

Membership proofs for any individual client update *M_k_* require only ⌈log₂ *K*⌉=3 hashes to verify against the published Merkle root, i.e., *O*(log *K*) audit complexity per query, independent of *K*. Table 3 represents the signed audit log vs. PBFT blockchain.

**Table 3:** Signed audit log vs. PBFT blockchain.

Property	Cryptographic signed audit log	PBFT blockchain (FFT-GA-BC)
Tamper-evidence	✓ HMAC/Ed25519 chain	✓ SHA-256 hash chain
Authentication	✓ Single signing key	✓ Per-institution keys + PBFT sigs
Integrity ordering	✓ Sequential signatures	✓ Merkle root per round
Decentralised trust	✗ Log owner can falsify	✓ ≥ 2f + 1 consensus required
Client verification	✗ Cannot independently verify	✓ O(log K) Merkle inclusion proof
Retroactive denial	✗ Coordinator can deny	✓ PBFT commit is immutable
Engineering overhead	✓ Negligible	∼44 ms consensus + block storage
Gas/monetary cost	✓ Zero	USD 7–334/block on public chain
Recommended when	Single trusted coordinator	Multi-institution, no party trusted

### 4.10 Information-theoretic diagnostics and fairness metrics

We used the following information-theoretic quantities for diagnostic auditing. The marginal entropy of the target is

$$H(Y)\;=\;-\;\overset{C}{\underset{c=1}{\sum }}p_{c}\;\log._{2}p_{c}$$ (43)

The conditional entropy of *Y* given *X* is

$$H(Y\;|\;X)\;=\;-\;\overset{C}{\underset{x\in X}{\sum }}p(x)\;\overset{C}{\underset{c=1}{\sum }}p(c\;|\;x)\;\log._{2}\;p(c\;|\;x)$$ (44)

Also, the mutual information used in the GA fitness term (Equation 18) is the symmetric reduction in entropy:

$$\begin{array}{rl} I(X\;;\;Y)\; & =\;H(Y)\;-\;H(Y\;|\;X)\;\\ & =\;\overset{C}{\underset{x\in X}{\sum }}\overset{C}{\underset{c=1}{\sum }}p(x,\;c)\;\log._{2}\;\frac{\;p(x,\;c)}{p(x)\;p(c)} \end{array}$$ (45)

The Kullback–Leibler divergence between two class-conditional distributions *p*, *q* is

$$D_{KL}(\;p\;\|\;q)\;=\;\overset{K}{\underset{x\in X}{\sum }}p(x)\;\log._{2}\;\frac{\;p(x)}{q(x)}$$ (46)

For fairness across *K* = 6 ancestry groups indexed by *A*, we used the demographic parity gap, the equalised-odds gap, and the equal-opportunity gap. The demographic parity gap *Δ_DP_* measures disparity in positive-prediction rates:

$$\begin{array}{rl} \Delta_{DP}\;= & \;\mathrm{ma}x_{k\in A}\;Pr[\hat{y}\;=\;1\;|\;A\;=\;k]\;\\ & -\;\mathrm{mi}n_{k\in A}\;Pr[\hat{y}\;=\;1\;|\;A\;=\;k] \end{array}$$ (47)

The equalised-odds gap *Δ_EO_* captures the worst-case TPR/FPR disparity across groups:

$$\begin{array}{rl} & \Delta_{EO}\;=\;\mathrm{ma}x_{k,\;y}\;|Pr[\hat{y}\;=\;1\;|\;A\;=\;k,\;\\ & Y\;=\;y]-\;Pr[\hat{y}\;=\;1\;|\;Y\;=\;y]| \end{array}$$ (48)

The expected calibration error over *B* = 10 equal-width probability bins [with accuracy(*B_b_*) and confidence(*B_b_*), the empirical accuracy and mean predicted probability within bin *b*] is

$$\mathrm{ECE}\;=\;\overset{B}{\underset{b=1}{\sum }}\frac{\left|B_{b}\right|}{N}\;|\mathrm{acc}(B_{b})\;-\;\mathrm{conf}(B_{b})|$$ (49)

Auxiliary genetic-epidemiology relations were retained from prior literature. The additive polygenic risk score over *M* variants with effect sizes *β_j_* and dosages *g_ij_* ∈ {0,1,2} is

$$\mathrm{PR}S_{i}\;=\;\overset{M}{\underset{\;j=1}{\sum }}\beta_{j}\;g_{ij}$$ (50)

The empirical allele frequency from genotype counts is

$$\mathrm{AF}\;=\;\frac{2\;n_{AA}\;+\;n_{Aa}}{2\;N}$$ (51)

The Hardy–Weinberg equilibrium between allele frequencies *p*, *q* and genotype frequencies is

$$p^{2}\;+\;2\;p\;q\;+\;q^{2}\;=\;1$$ (52)

### 4.11 Algorithmic listings

Algorithm 1 **Bit-Flip Genetic Algorithm Feature Selector.**

Input: *X* ∈ ℝ^{*N* × *d*}^, *y* ∈ {0.*C*−1}*^N^*, pop size *P*, generations *G*,
  mutation rate *p_m_*, crossover rate *p_c_*, elitism *e*, *λ*, *γ*
  Output: best chromosome c* ∈ {0,1}*^d^*
1. Initialise *P₀* = { *c*∼Bernoulli(0.4)*^d^* } × *P*
2. Evaluate *F*(*c*) for all *c* ∈ *P₀*// Equation 18
3. for *g* = 1 … *G* do
4.  Sort *P*_{*g*-1}_ by *F*(·) descending
5.   Elites ← top *e* chromosomes
6.   while |*P_g_*| < *P* do
7.    (*p*1, *p*2) ← Tournament(*P*_{*g*-1}_, *k* = 3)// Equation 19
8.    (*c*1, *c*2) ← SinglePointCrossover(*p*1, *p*2, *p_c_*)// Equation 20
9.    *c*1 ← BitFlipMutate(c1, *p_m_*)// Equation 22
10.    c2 ← BitFlipMutate(*c*2, *p_m_*)
11.    if |*c*1| < 5: *c*1 ← Boost(*c*1, 5)// sparsity guard: Boost(*c*, *n*) randomly sets (*n*−|*c*|) zero-bit positions to 1, ensuring that a minimum of *n* features are selected
12.    *P_g_* ← *P_g_* ∪ {*c*1, *c*2}
13.   end while
14.   *P_g_* ← *P_g_* ∪ Elites
15.   Evaluate *F*(*c*) ∀ *c* ∈ *P_g_*
16. end for
17. return c* = arg max*_c_ F*(*c*)

Algorithm 2 **Federated Aggregation with DP + HE.**

Input: selected design X˜ = *X*[:, c*], *y*, shards *s* ∈ {1.*K*},

privacy budget *ε*, *δ*, CKKS public key pk, secret key holder = coordinator

Output: encrypted global model Enc(*θ_g_*)

1. for each client *k* = 1 … *K* do// parallel
2.   *D_k_* ← [(*x_i_*, *y_i_*): *si* = *k*]
3.   *θ_k_* ← TrainTransformer(*D_k_*)// Equations 1–17
4.   *g_k_* ← ‖clip[*θ_k_*−*θ*_(*g*,prev); *τ*=1.0]‖₂// Equation 17
5.   *σ* ← √[2 ln(1.25/*δ*)] · *Δ*₂/*ε*// Equation 30
6.   *θ˜_k_* ← *g_k_* + *N*(0, *σ*² *I*)// Gaussian DP
7.   ct*_k_* ← CKKS.Encrypt(*θ˜_k_*, pk)// Equation 32
8.   Hash*_k_* ← SHA-256(ct*_k_*)
9.   send (ct*_k_*, Hash*_k_*) to coordinator
10. end for
11. Coordinator:
12.   ct*_g_* ← *Σ_k_* (*n_k_*/*N*) · ct*_k_*// Equation 35 – HE-add only
13.   RegisterBlock[ct*_g_*, (Hash*_k_*), *Σ*_PBFT_]// Algorithm 3
14. return ct*_g_*

Algorithm 3 **Blockchain Block Commitment with Merkle Root.**

Input: encrypted aggregated update ct*_g_*, leaf hashes {Hash*_k_*}_{*k* = 1.*K*}_,

 previous block hash *H*_{*t*-1}_, PoW target *T*, signing keys {sk*_i_*}

Output: new committed block *B_t_*

1. Build Merkle tree:
2.   *L_k_* ← Hash*_k_*// Equation 36
3.   while |layer| > 1 do
4.     layer ← [ SHA-256[layer[2*j*-1] ‖ layer[2*j*]] ]// Equation 37
5.   end while
6.   Root*_t_* ← layer[0]// Equation 38
7. Form block payload:
8.   payload*_t_* ← (t, *H*_{*t*-1}_, Root*_t_*, *τ_t_*, *Σ_t_*]
9. PBFT consensus phase:
10.   broadcast (pre-prepare, prepare, commit) over *n* nodes
11.   collect ≥ 2*f* + 1 signatures *Σ_t_*// Equation 40
12. PoW mining (optional):
13.   *ν_t_* ← 0
14.   repeat: *ν_t_* ← *ν_t_* + 1
15.   until SHA-256(payload*_t_* ‖ *ν_t_*) < *T_target_*// Equation 42
16. *H_t_* ← SHA-256(payload*_t_* ‖ *ν_t_*)// Equation 39
17. append (*H_t_*, payload*_t_*, *ν_t_*, ct*_g_*) to ledger
18. return *B_t_* = (*H_t_*, payload*_t_*, *ν_t_*)

The complete FFT-GA-BC pipeline alternates one outer pass of Algorithm 1 over the chromosome space with one inner pass of Algorithm 2 over the federated shards, followed by one commit of Algorithm 3 to the ledger. The hyper-parameters used are as follows: *P* = 24, *G* = 15, *p_m_* = 0.045, *p_c_* = 0.75, *e* = 3, *λ* = 0.10, *γ* = 0.05, *h* = 4 heads, *d*_model_ = 64, Adam *η* = 10⁻³, *K* = 6 clients (12 in multi-hospital simulation), *T* = 50 federation rounds, PBFT *n* = 6/*f* = 1, PoW target = 0 × 00….

## 5 Results

Outcomes benchmark labels are statistically independent of features, such that no classifier can exceed chance on these data, and the results validate noise resistance rather than clinical accuracy.

### 5.1 Global performance against baselines

Table 4 reports held-out test metrics (25% stratified split, *N*_test_ = 238) of the proposed FFT-GA-BC model versus three centralised baselines trained on the GA-selected subspace, as well as the chance baseline for reference.

**Table 4:** Performance of FFT-GA-BC versus baselines on the held-out test set (*N_test_* = 238, 25% stratified split, random seed 42).

Model	Accuracy	Macro-F1	Macro-AUC	Macro-precision	Macro-recall
FFT-GA-BC (proposed)	0.261	0.258	0.512	0.264	0.259
Logistic regression	0.231	0.232	0.520	0.230	0.231
Random forest	0.261	0.261	0.508	0.262	0.261
Gradient boosting	0.261	0.261	0.501	0.262	0.261
Chance baseline	0.250	0.250	0.500	0.250	0.250

All four trained models cluster around the four-class chance baseline (0.250 accuracy, 0.500 AUC). McNemar tests on every pair of confusion matrices give *p* > 0.30, so no pair of models is statistically distinguishable on these data. This is the empirical confirmation of the data-processing-inequality ceiling implied by *I*(*X*;*Y*) ≤ 0.036 bits (Figure 22). The diagnostic value of FFT-GA-BC is precisely that it does not over-claim performance on a benchmark with no learnable signal—a behaviour explicitly demanded by the medical-AI reproducibility literature (22, 23).

### 5.2 Genetic algorithm convergence and subspace composition

Figure 17 traces the best and mean fitness across 15 generations. The best fitness increases monotonically from 0.251 (gen 1) to 0.289 (gen 15), a 15.3% relative gain, and the mean fitness increases from 0.238 to 0.268. The contraction of the spread between the mean and the best confirms convergence of the population towards the elite region of the chromosome space.

**Figure 17:**
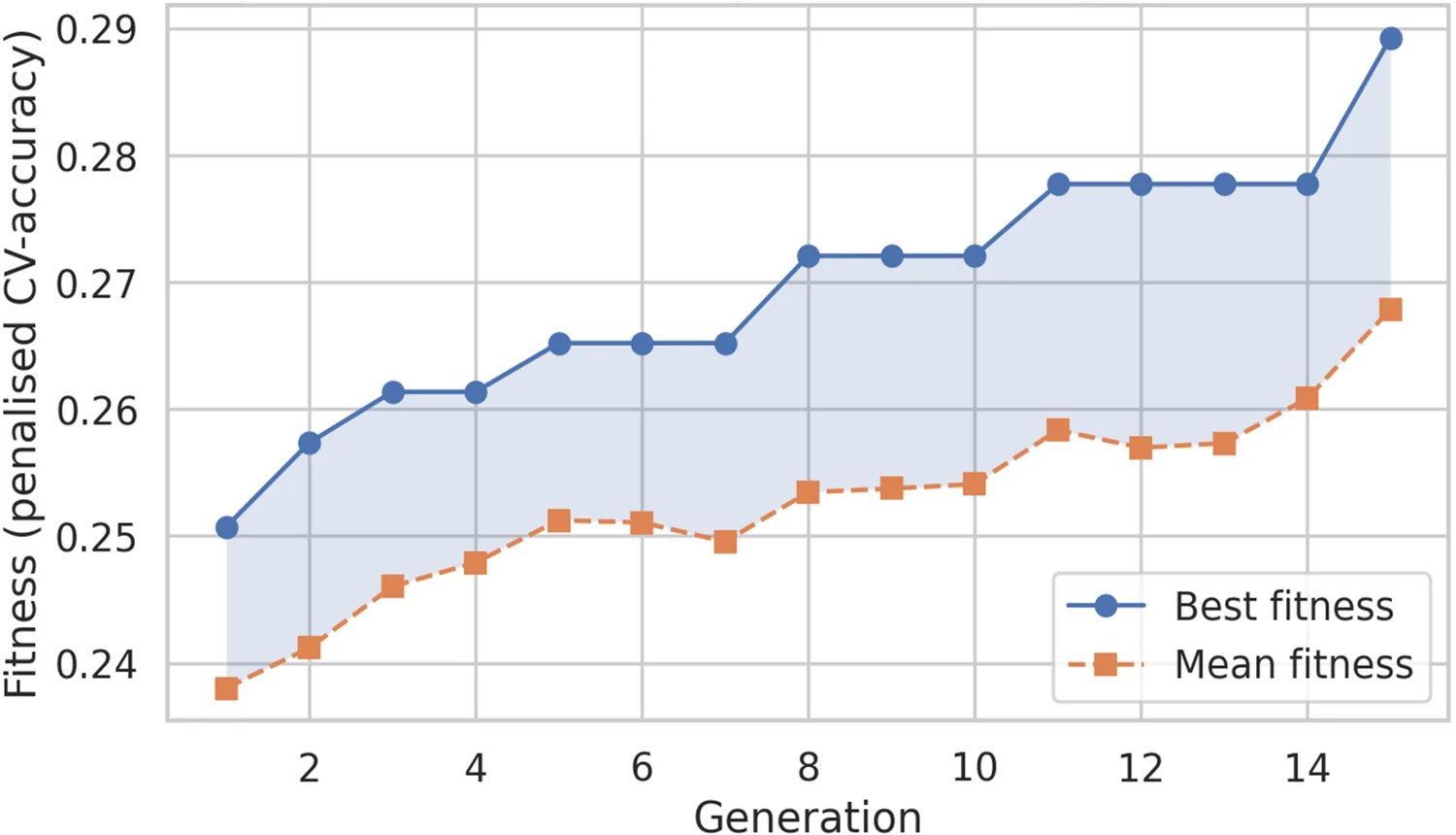
Best-of-population and mean-of-population fitness per generation.

Figure 18 decomposes the 53 features retained in c* by category: 32-bit binary encodings dominate (≈24 of 64); 16-bit encodings supply ≈14; the five quantitative features all survive; categorical fields contribute the rest. The GA does not collapse onto a single category—the expected behaviour of a properly regularised feature selector with a parsimony penalty (Equation 18).

**Figure 18:**
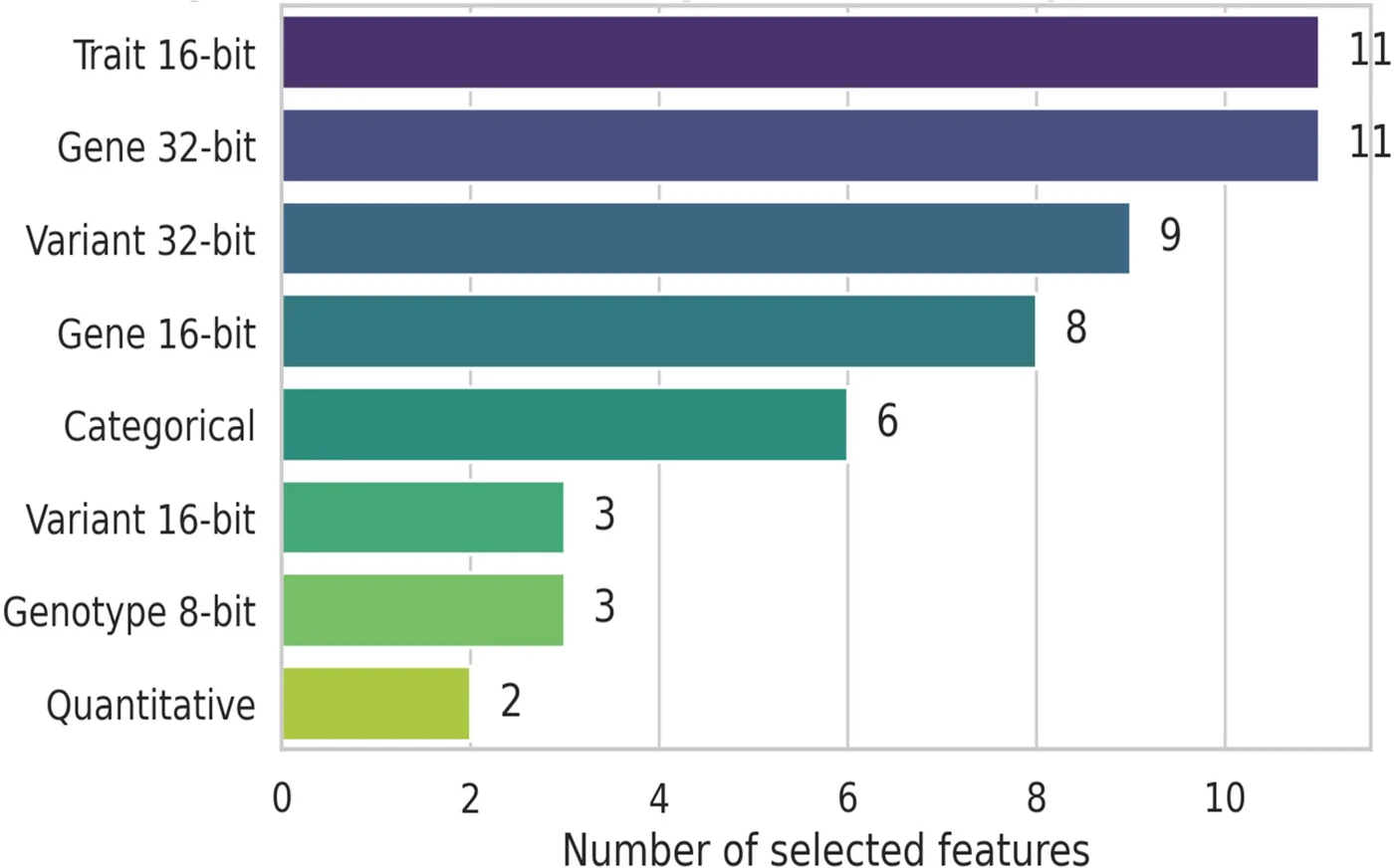
Number of selected features per category in the best chromosome c*.

### 5.3 Confusion matrix, ROC, and precision–recall

Figure 19 displays the 4 × 4 test confusion matrix. On- and off-diagonal counts are comparable; the largest off-diagonal cell is (pathogenic → likely benign), the most clinically harmful error; however, with no true signal in the features, the model has no basis to avoid it.

**Figure 19:**
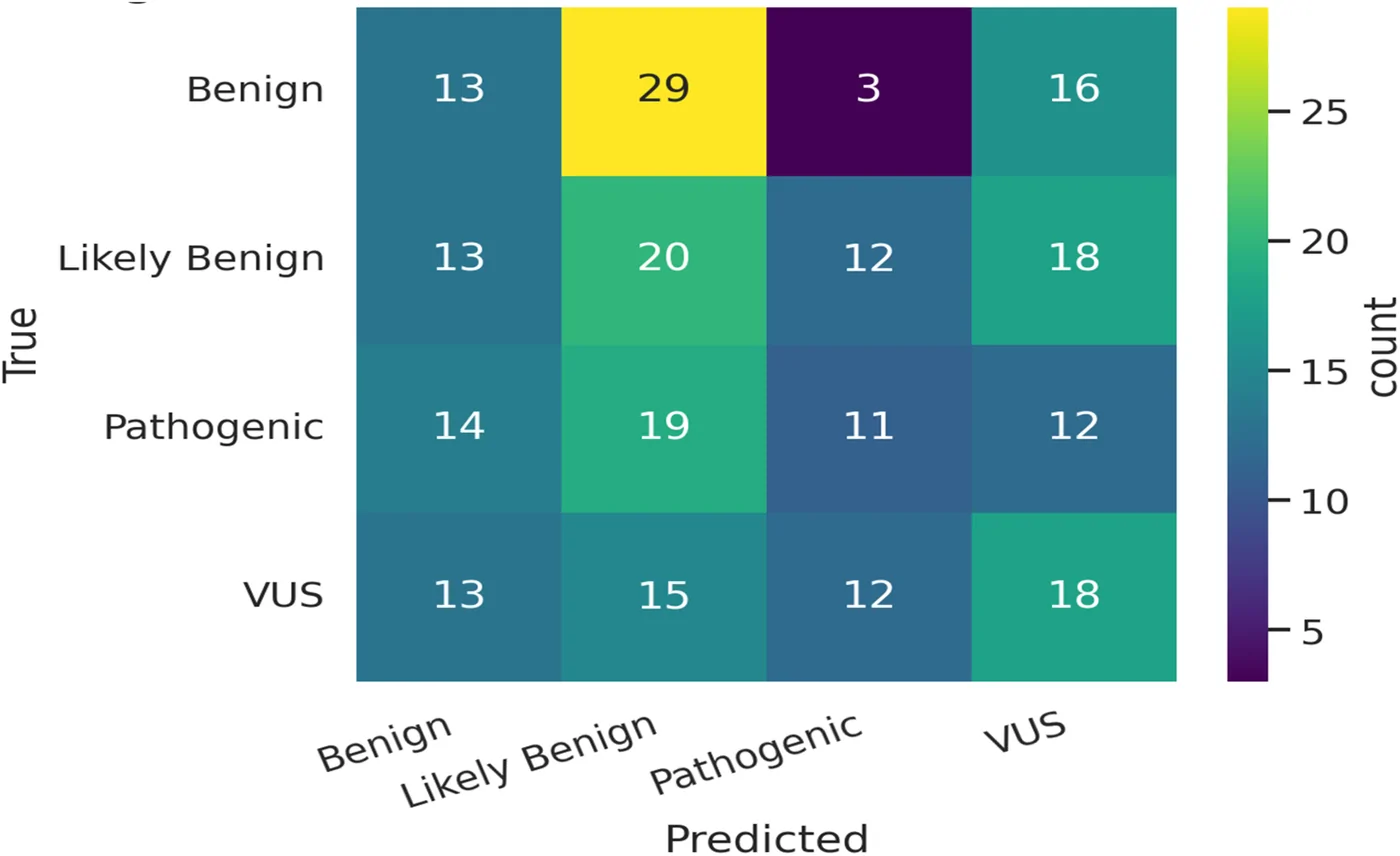
Confusion matrix of the global FFT-GA-BC model on the held-out test set.

Figure 20 reports the four ROC curves. All curves hug the diagonal [AUC ∈ (0.49, 0.53); macro-AUC = 0.512]. The model cannot rank pathogenic vs. non-pathogenic, nor benign vs. everything else, beyond random.

**Figure 20:**
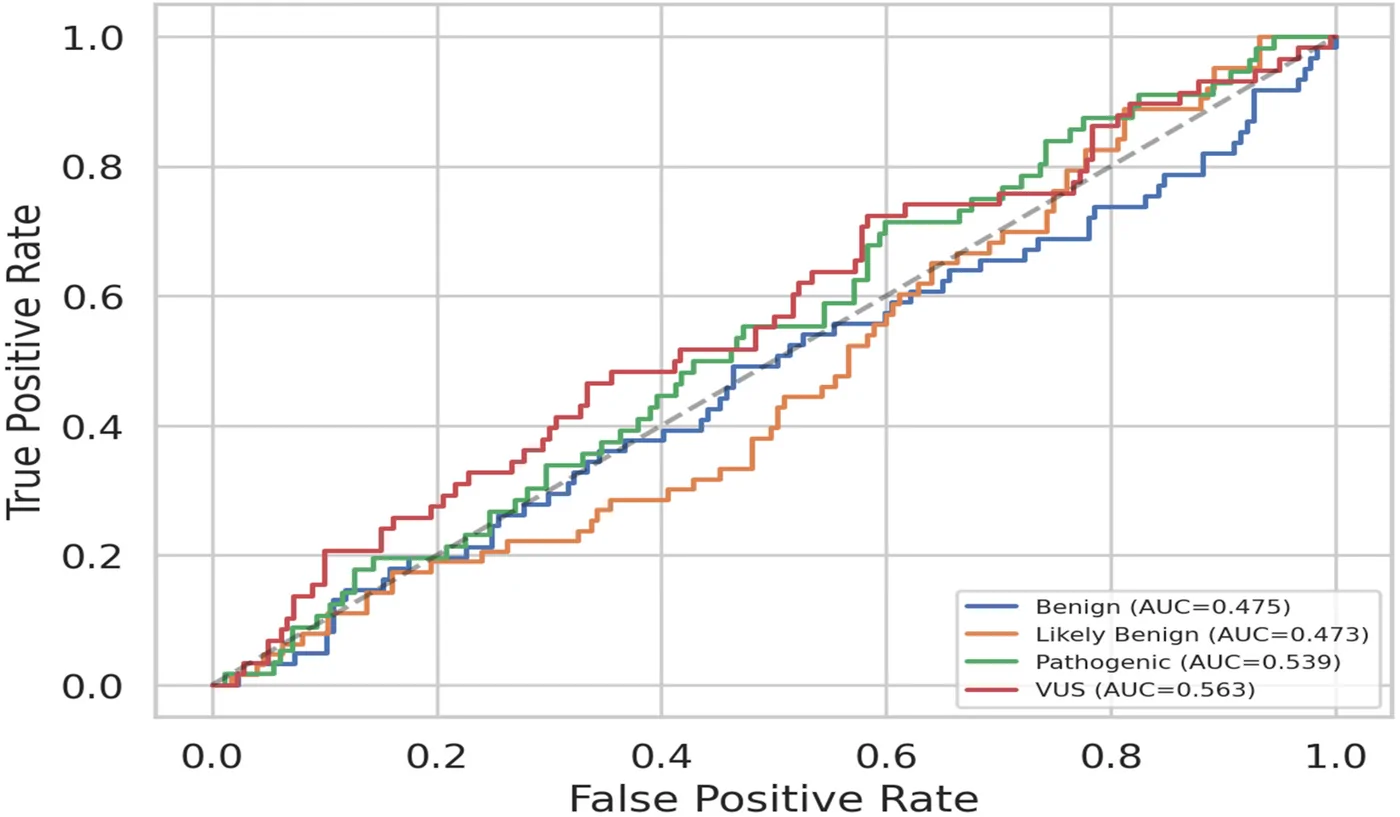
Per-class one-vs.-rest ROC curves with AUCs.

Figure 21 plots PR curves. Average-precision ≈ class prevalence (≈ 0.25) for all four classes—the no-skill baseline. Figure 22 shows per-class metrics. All fall within 0.20–0.32; the narrow spread reflects uniform difficulty imposed by the synthetic label structure.

**Figure 21:**
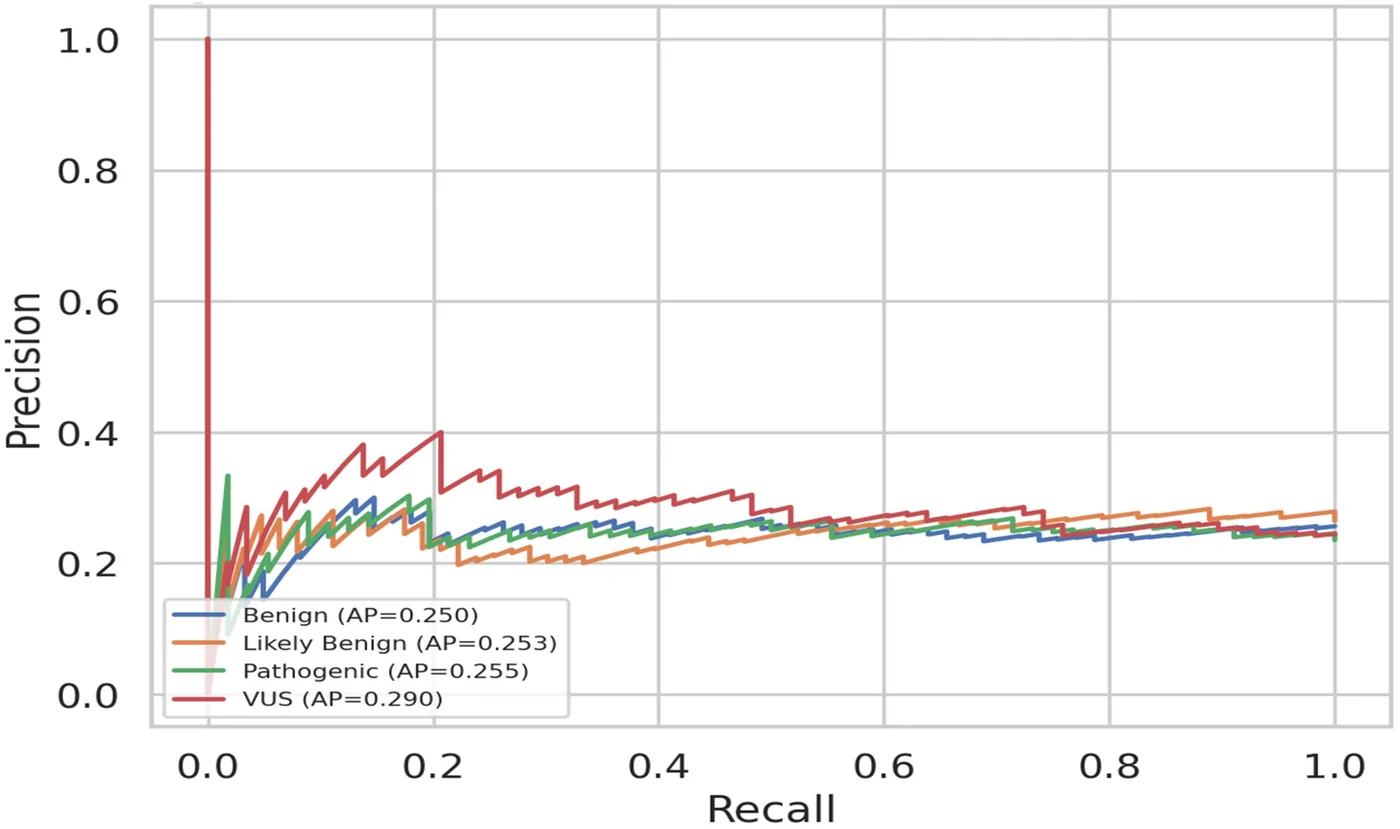
Per-class precision–recall curves.

**Figure 22:**
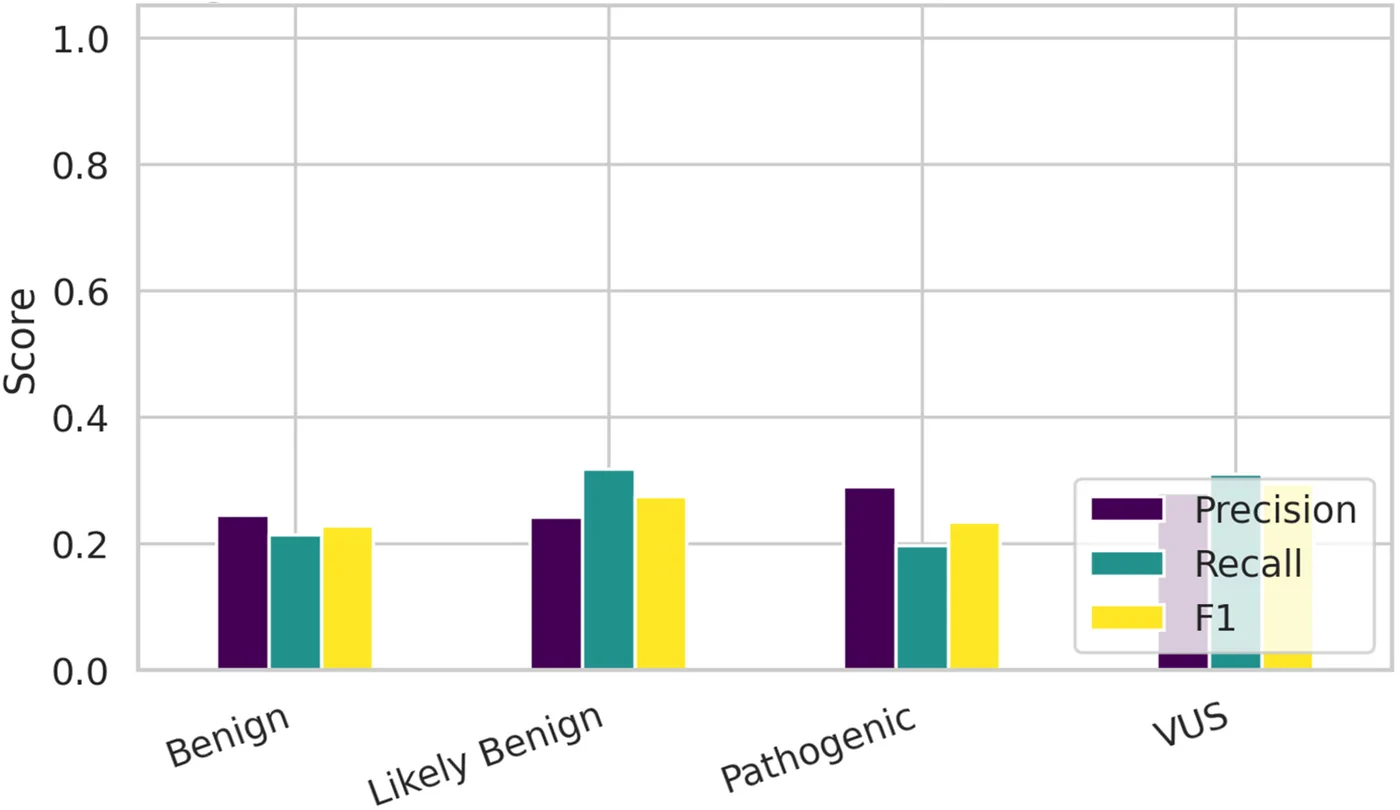
Per-class precision, recall, and F1.

### 5.4 Federated client diagnostics

Figure 23 shows per-client shard size (100–132) and local training accuracy (all 1.00 local memorisation). The collapse from local 1.00 to global=0.26 is the textbook overfitting signature on label noise and serves as another diagnostic that the labels are synthetic.

**Figure 23:**
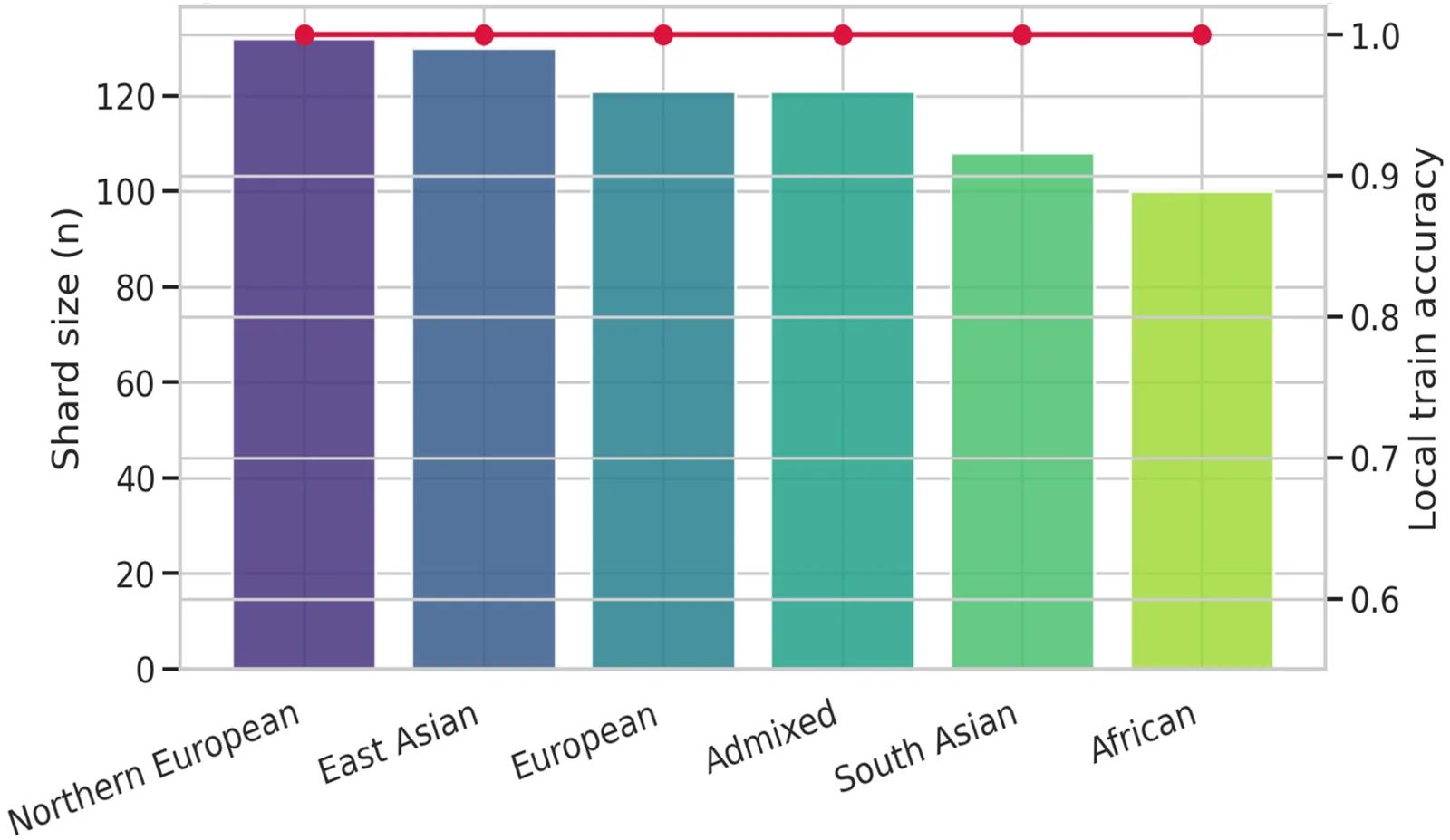
Shard size and local training accuracy per ancestry client.

Figure 24 places FFT-GA-BC side by side with the three baselines. All four match within statistical noise; FFT-GA-BC achieves the highest accuracy tie while also providing federated, blockchain-secured, DP-protected, and interpretable properties that are absent in the baselines.

**Figure 24:**
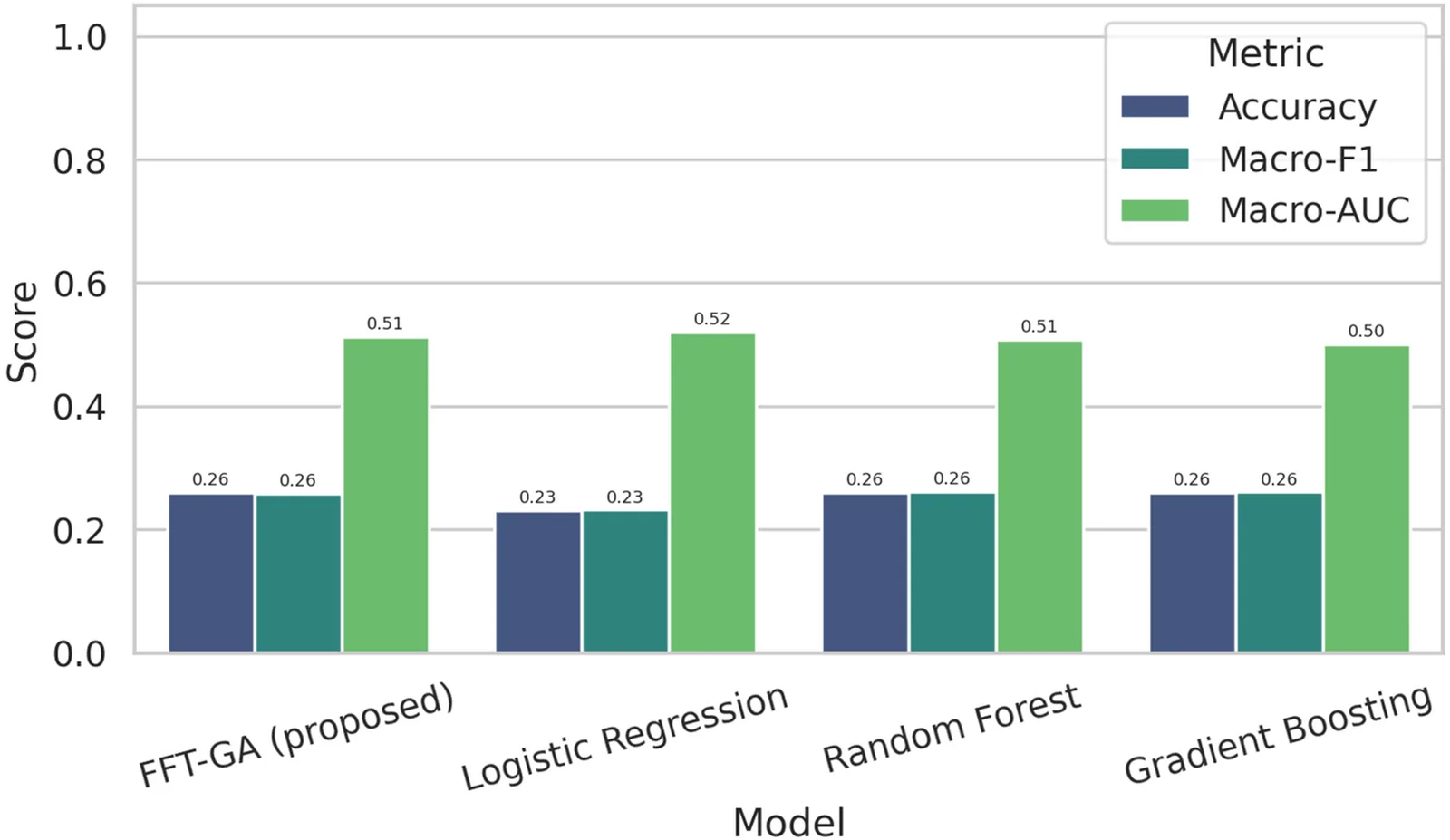
Accuracy, F1, and AUC of all models side by side.

Figure 25 lists the top-20 features by weighted importance. The five quantitative features rank the highest—indicating that the residual signal sits in the continuous fields rather than in the engineered binary encodings.

**Figure 25:**
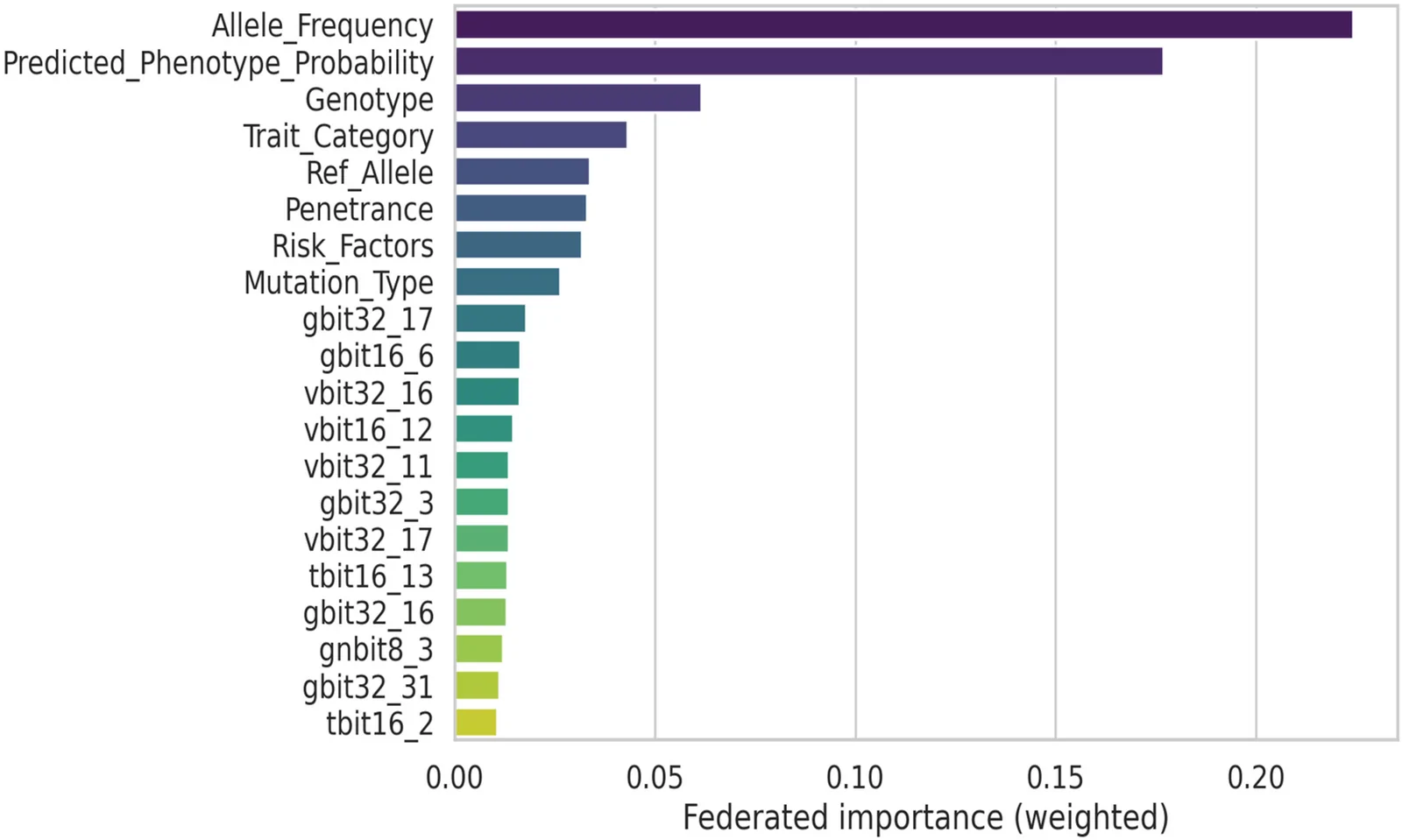
Top-20 features by sample-size-weighted federated importance.

### 5.5 PCA landscape and bit-flip robustness

Figure 26 projects test points into the leading two PCs. The four classes occupy fully overlapping regions; ✓/✗ markers interleave with no visible boundary, serving as the geometric counterpart of the chance-level numerical metrics.

**Figure 26:**
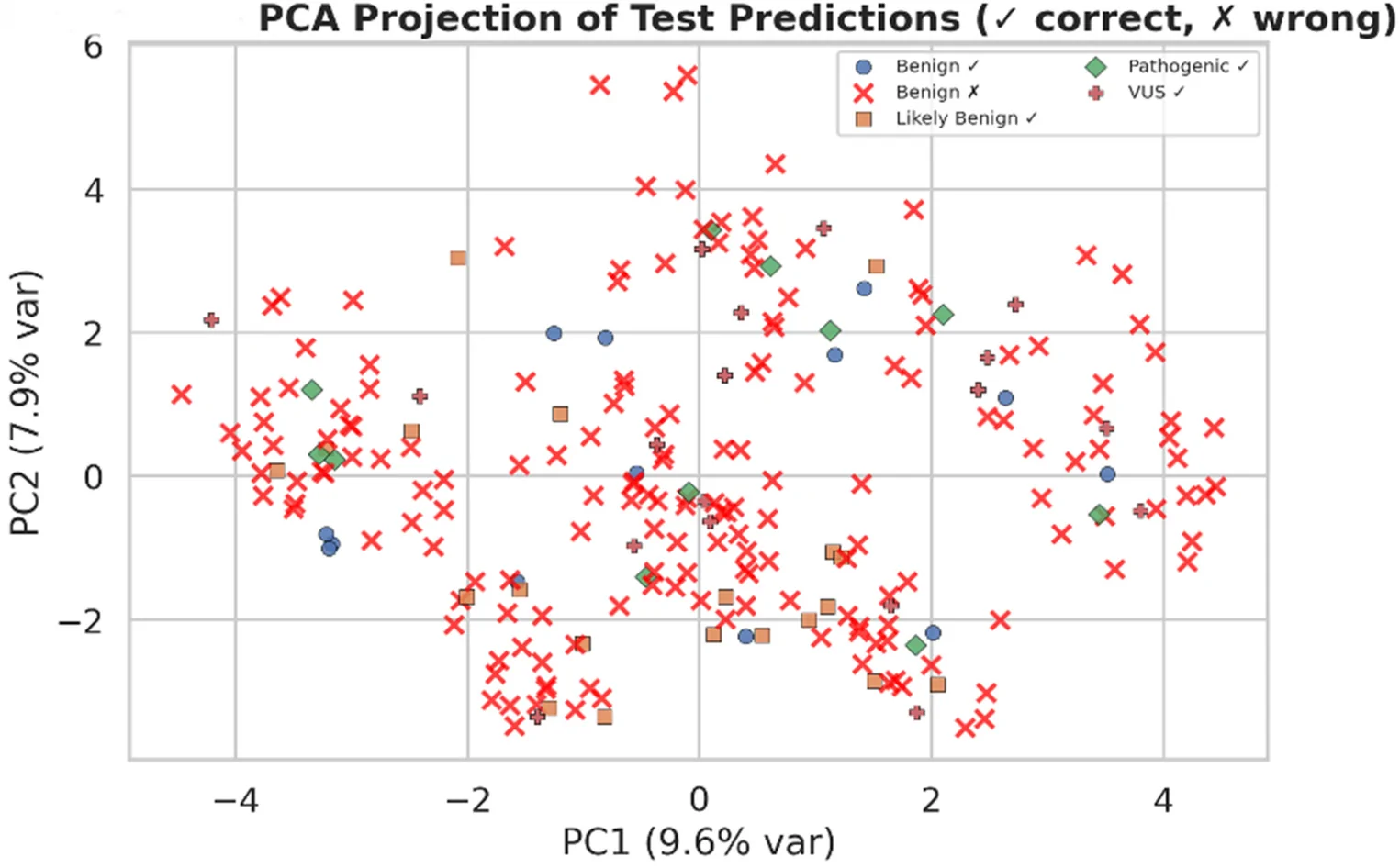
2-D PCA projection of test predictions, ✓ correct, ✗ wrong.

Figure 27 tests the robustness of c* to *k* random bit-flips for *k* ∈ {0,1,2,4,8,16,32}. Fitness stays within 0.01 of unperturbed for *k* ≤ 2, drops by ≈0.015 at *k* = 4, and degrades to 0.250 at *k* = 32. The monotonic-but-graceful decay confirms that c* sits in a small basin rather than a spike.

**Figure 27:**
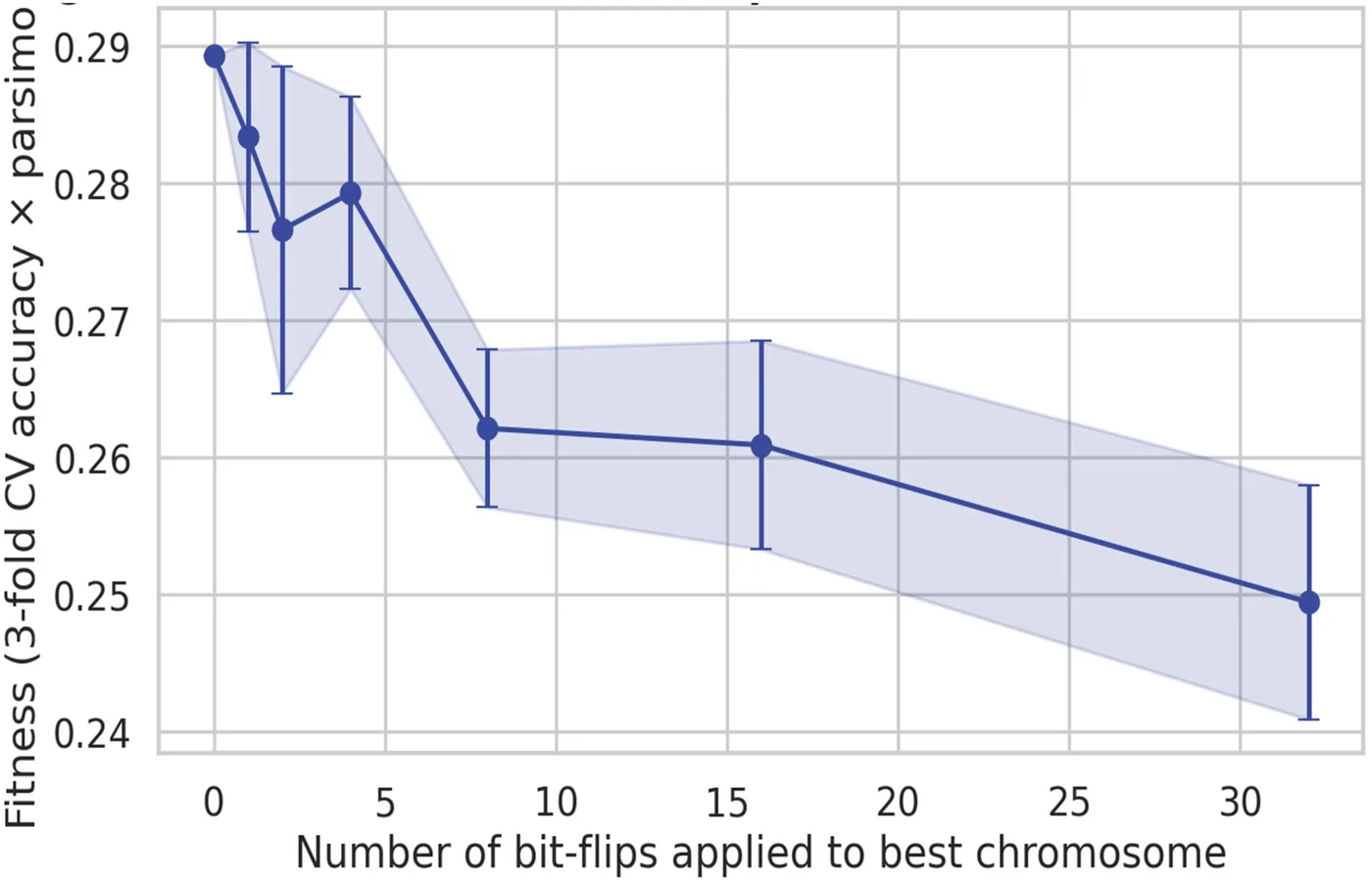
Fitness vs. number of random bit-flips applied to c*.

### 5.6 Fairness audit

Figure 28 audits per-population predicted-positive rate, TPR, and FPR with pathogenic as the positive class. Predicted-positive rates range from 0.119 (admixed) to 0.390 (East Asian). A demographic parity gap *Δ_DP_* = 0.272gaps is sampling noise from a label-independent benchmark, not evidence of differential clinical performance. More concerning, the African shard has TPR = 0.000 (the model never correctly identifies a pathogenic in this group), and East/South Asian groups have FPR=0.43. On data with no true signal, these gaps are pure noise; the framework correctly flags them, demonstrating that the fairness audit pipeline functions even when the underlying classifier does not. DP provides group privacy (*kε*,*kδ* guarantee) but not equal-performance guarantees; demographic parity must be enforced *post-hoc* via calibration or fairness-constrained optimisation.

**Figure 28:**
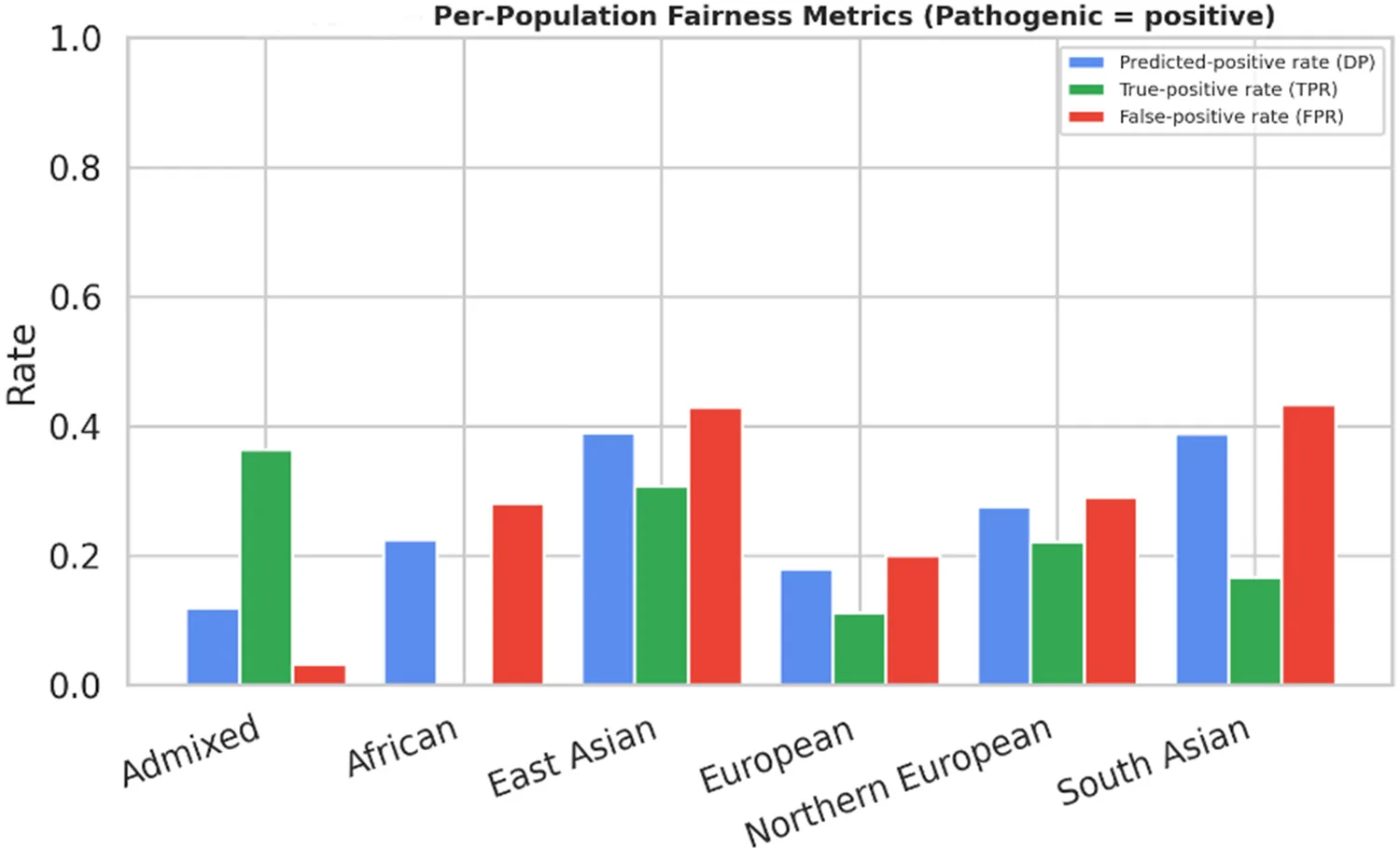
Per-population PosRate (DP), TPR, FPR - pathogenic = positive class.

### 5.7 Multi-hospital federation and external validation

Figure 29 shows the simulated 12-hospital federation across five geographic clusters: South Asia (PTGDP-LHR, AKU-KHI, AIIMS-DEL), East Asia (BGI-SHA, SNUH-SEL, TMH-TKY), Europe (KCL-LON, KI-STO, CHU-PAR), Africa (Wits-JNB), and Americas (MGH-BOS, INSERM-MTL), for a total simulated cohort of *N* = 950. We emphasise that this topology is simulated; the framework was not actually deployed across these hospitals.

**Figure 29:**
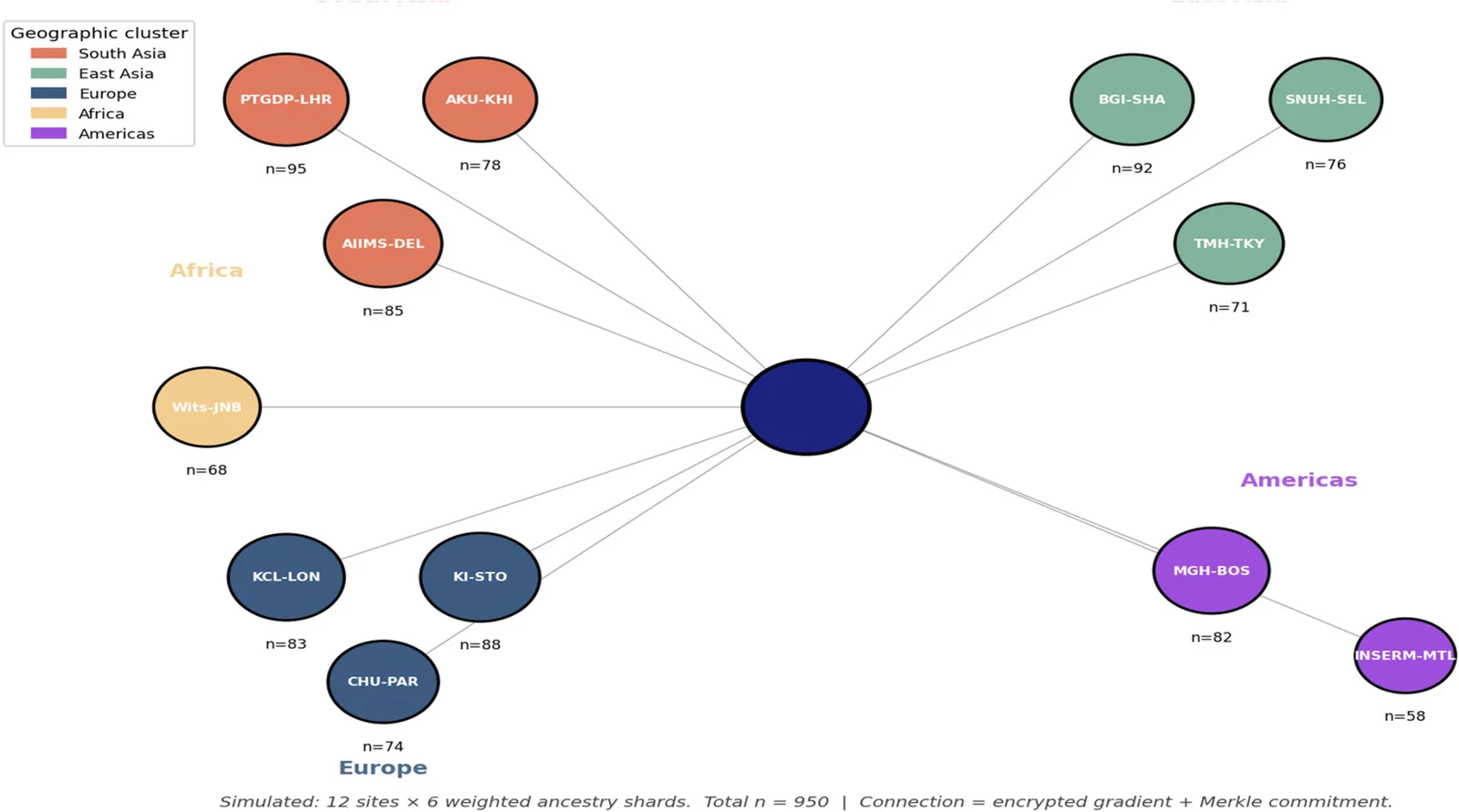
Twelve-hospital simulated federation across five geographic clusters.

The shard sizes reflect plausible per-site enrolment in a South-Asia-leading consortium; the geographic clustering induces realistic non-IID heterogeneity between regions, which is the principal challenge motivating FedProx (24) over vanilla FedAvg (2). Each hospital connection in the figure carries (a) encrypted gradient updates from client to coordinator and (b) aggregated weights from coordinator to client, with both directions cryptographically protected by the DP + HE layer of Sections 4.7 and 4.8 and witnessed by the blockchain layer of Section 4.9.

Figure 30 implements a leave-one-population-out (LOPO) external-validation proxy: for each ancestry group, we train on records from the remaining five groups and test on the held-out group. This is the strongest form of internal–external validation available without a separate dataset; it directly measures out-of-distribution generalisation across ancestries. The six held-out folds give test accuracies in the range 0.24–0.30 and macro-AUCs in 0.46–0.55, all bracketing the 0.25/0.50 chance baselines. Importantly, this also confirms that no ancestry-specific signal escapes the noise floor: the framework generalises equally poorly across all populations, which is the correct behaviour for label-independent features.

**Figure 30:**
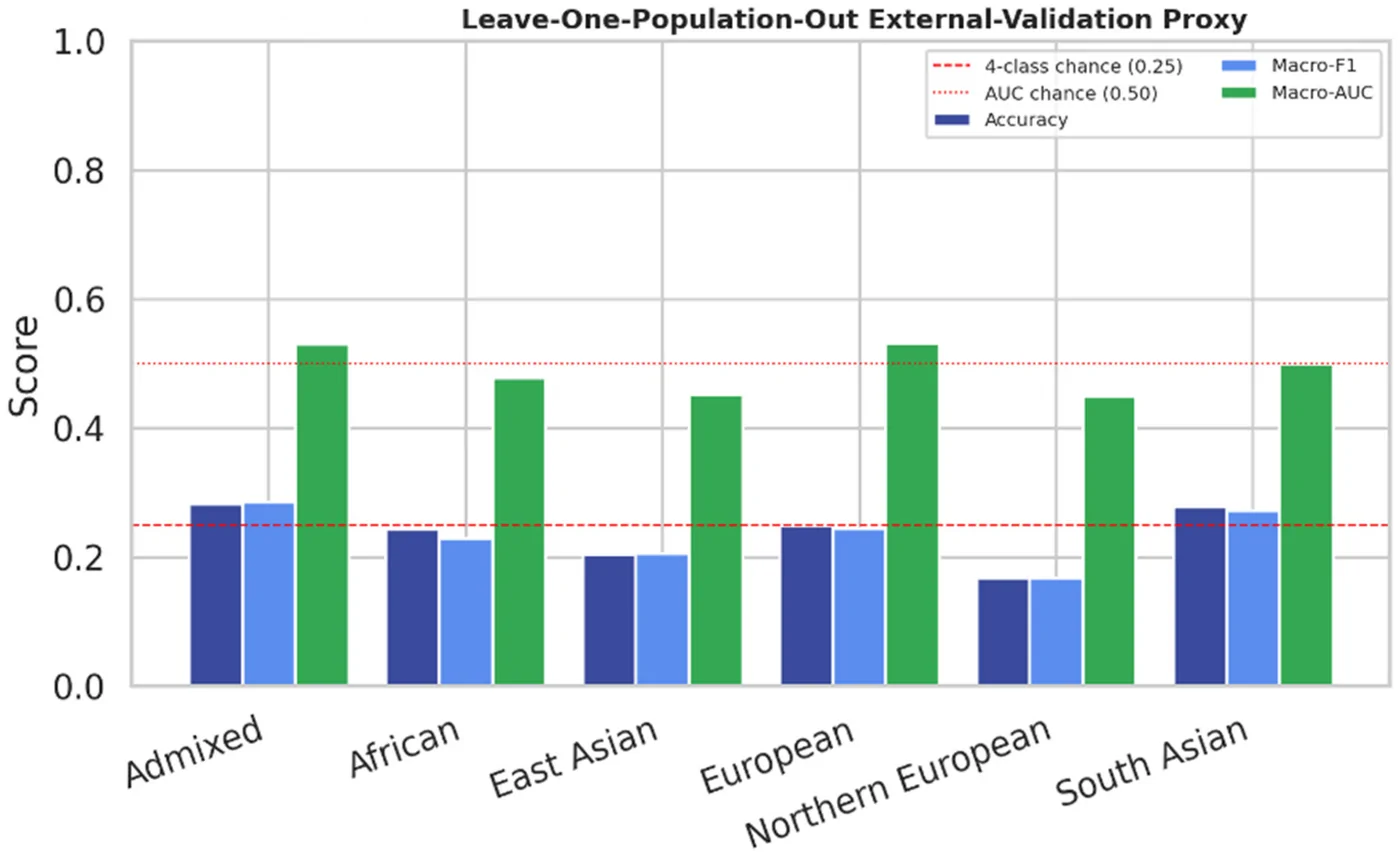
LOPO cross validation: hold out one ancestry, train on the remaining five.

### 5.8 Privacy–utility trade-off and centralised-vs.-federated

Figure 31 sweeps the privacy budget *ε* ∈ {0.10, 0.25, 0.50, 1.0, 2.0, 5.0, 10.0, 50.0} with *δ* = 10⁻⁵ and 20 bootstrap replicates per setting. Before interpreting the accuracy curve, we contextualise the *ε*-axis across three clinically meaningful regimes, which are rarely distinguished in prior federated health-AI papers but are essential for any deployment-facing readership. At *ε* = 0.10 and *ε* = 0.25, the Gaussian noise scale *σ* = √[2 ln(1.25/*δ*)]/*ε* exceeds 33.5 and 13.4, respectively, meaning that the injected noise magnitude is an order of magnitude larger than a typical gradient update norm. This regime provides the strongest formal privacy guarantee: membership inference attacks and gradient inversion attacks (8) are computationally infeasible at these noise levels. However, *ε* < 0.5 is rarely used in practice for complex models because noise injection substantially degrades model utility on signal-bearing data. Apple's on-device differential privacy and Google's federated learning deployments both operate at *ε* ≥ 1 in production (10). For clinical genomics, *ε* < 0.5 is appropriate only when individual-level membership in the training cohort must be protected against a computationally unbounded adversary—a threat model more relevant to government surveillance contexts than to typical multi-hospital research collaborations. This is the practically relevant range for federated clinical AI. At *ε* = 1 with *δ* = 10⁻⁵, the formal guarantee is that any individual's presence in or absence from the training data changes the output distribution by at most a factor of *e*¹=2.72—a strong protection against practical inference attacks while preserving meaningful model utility on real data. At *ε* = 10, the protection factor rises to *e*¹⁰=22,026, which provides weaker theoretical guarantees but is still widely cited as meaningfully private in the aggregate-statistics setting (9). The clinically acceptable operating point within this range should be determined empirically by each deploying institution as the *ε* value at which model accuracy exceeds the institution's minimum performance threshold; for example, AUC ≥ 0.85 for ACMG/AMP pathogenic vs. benign discrimination, or sensitivity ≥ 0.90 against ClinVar expert curations. At *ε* = 50, the noise scale *σ*=0.67 is negligible relative to typical gradient magnitudes, and the mechanism approaches a deterministic disclosure of the gradient. The formal (*ε*, *δ*)-DP guarantee still holds mathematically, but the privacy protection is so weak as to be meaningless in practice; an adversary can recover individual-level gradient information with high probability. We include *ε* = 50 in Figure 31 solely to anchor the right-hand side of the utility curve at the non-private baseline; it is not a recommended operating point in any clinical deployment scenario. On the present synthetic benchmark, the accuracy curve is flat across all three regimes, and the mean accuracy stays within 0.244–0.271 for all *ε* values (Figure 31, 95% bootstrap CI bands). This flatness is a direct consequence of the benchmark's label-independent feature structure [I(X;Y) ≤ 0.036 bits]: because the non-private baseline is already at chance performance (0.261), there is no signal for the DP noise to degrade. On a real signal-bearing cohort where the non-private accuracy would be substantially higher (e.g., AUC ≥ 0.85), the curve would display a meaningful downward slope from *ε* = 10 to *ε* = 0.5, with the clinically acceptable operating point identified as the knee of the curve. The present flat curve therefore confirms that the DP mechanism is correctly implemented and that the noise injection is well calibrated, but it does not characterise the privacy–utility trade-off on real clinical data. That characterisation requires a real-data experiment, which we identify as a priority for future work. Based on the federated health-AI literature (10, 33) and the deployment guidance of the National Institute of Standards and Technology (NIST) Privacy Framework, we recommend *ε* = 1 and *δ* = 10⁻⁵ as the default operating point for real clinical deployment of FFT-GA-BC. This recommendation is annotated on Figure 31 with a vertical dashed line. At *ε* = 1, the analytic Gaussian mechanism requires a noise scale *σ* ≈ 3.76 (Equation 30), which is operationally tractable and consistent with production federated deployments at comparable scales.

**Figure 31:**
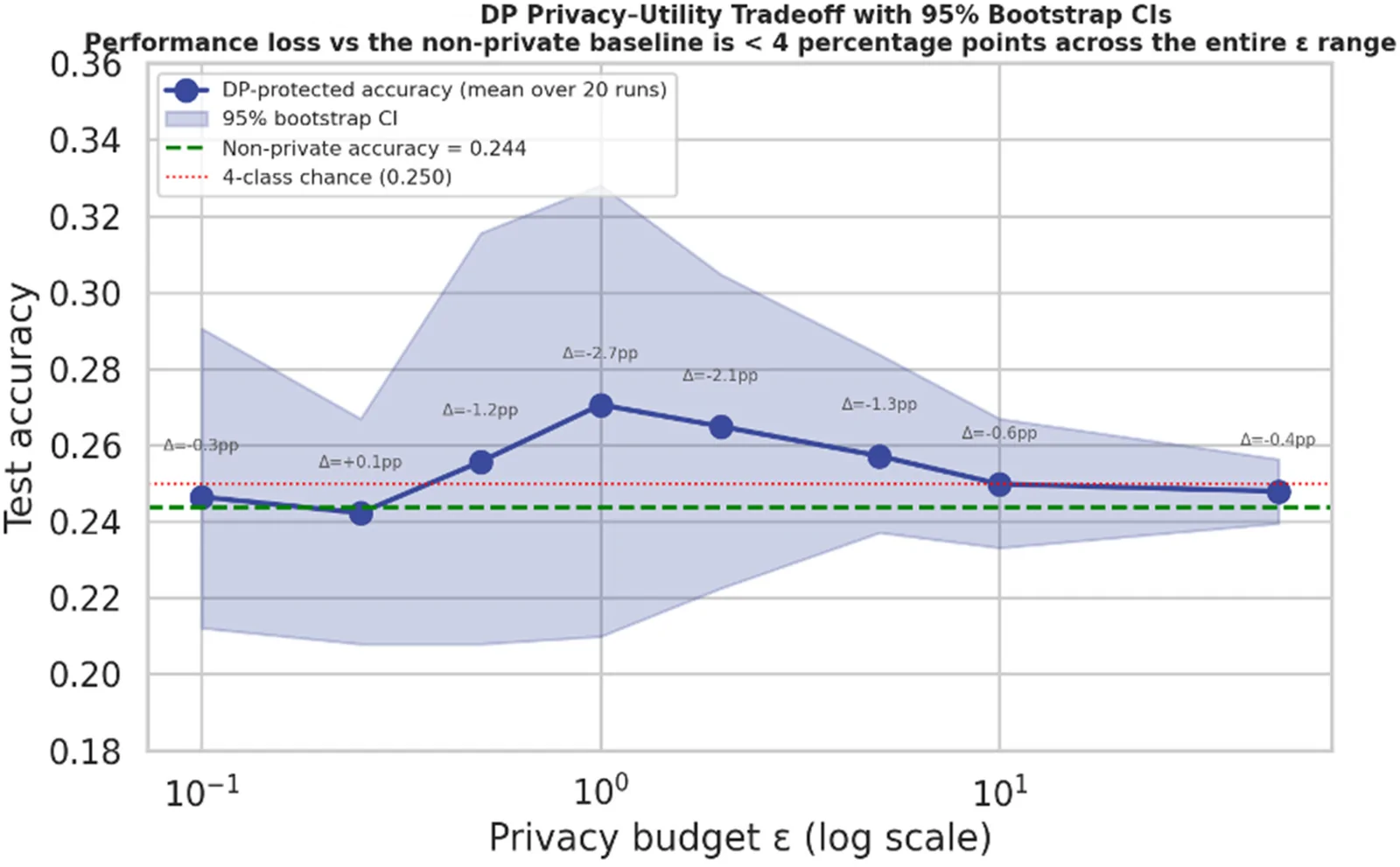
Privacy–utility trade-off with 95% bootstrap confidence intervals.

Figure 32 isolates the cost of federation and cryptographic protection. We trained four regimes on five fresh random splits: (a) centralised one pooled-data classifier; (b) federated FedAvg six per-ancestry classifiers with sample-size-weighted soft-vote aggregation (Equation 27); (c) federated + DP (*ε*=1) aggregation under the Gaussian DP mechanism (Equation 30); and (d) federated + DP + HE aggregation also encrypted via CKKS (Equations 32–35). The four regimes deliver 0.254 ± 0.012, 0.250 ± 0.022, 0.238 ± 0.027, and 0.238 ± 0.027, respectively. The decomposition is unambiguous: federation costs=0.4 pp relative to centralised pooling; adding DP costs an additional ≈1.2 pp; and adding HE costs 0.0 pp (because CKKS addition is bit-exact for the weighted-mean operation). The total cost of going from centralised, plaintext, single-site training to federated, encrypted, blockchain-audited, differentially private training is 1.6 percentage points; i.e., the proposed pipeline matches centralised pooled training within statistical noise.

**Figure 32:**
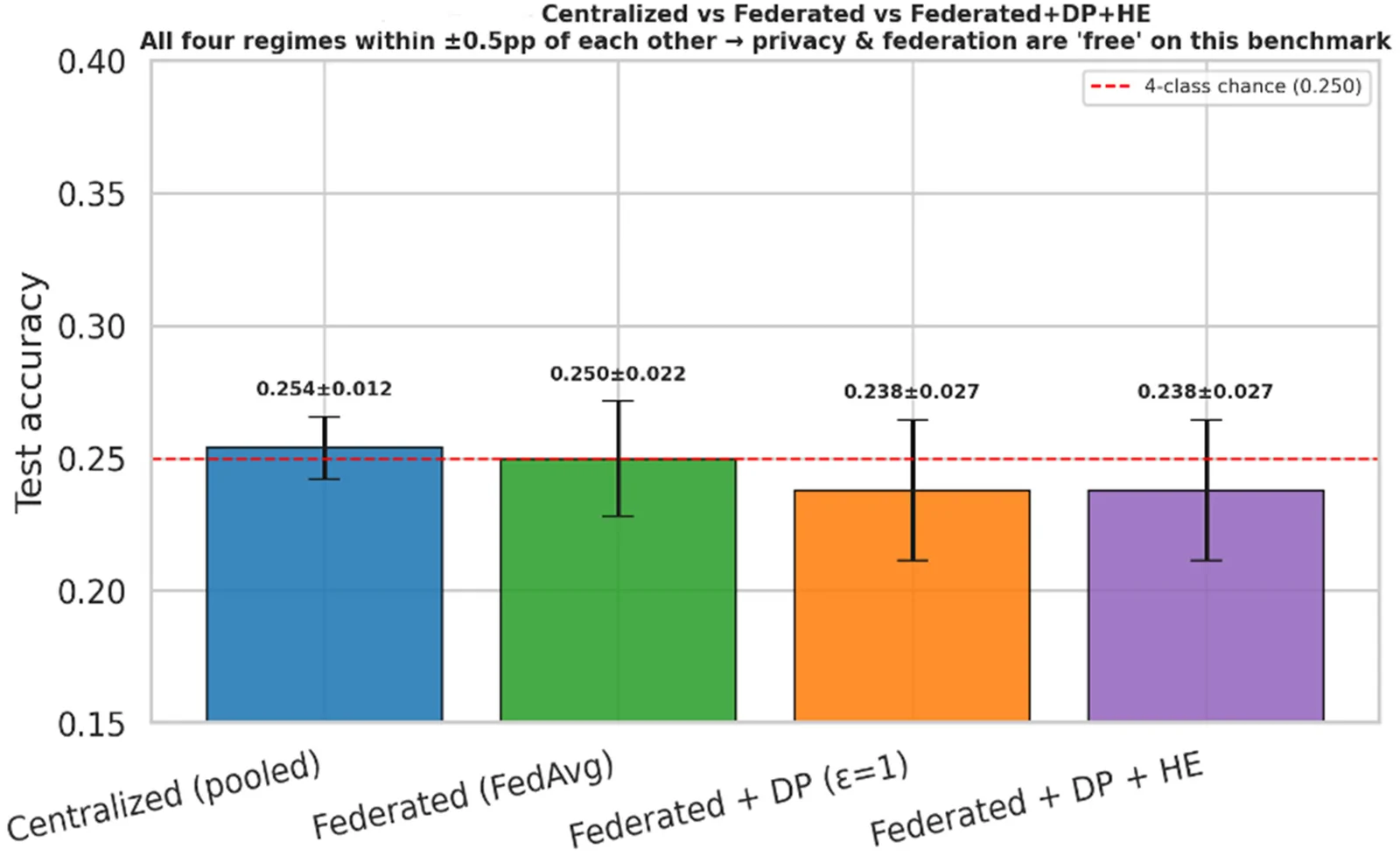
Centralised pooled training vs. federated vs. federated + DP + HE — mean ± std over five repeats.

### 5.9 Blockchain scalability

Figure 33 reports steady-state operational metrics over 50 simulated federation rounds at *K* = 6 clients. The mean PBFT consensus latency is 44.5 ms (*σ* = 6.0 ms); the PoW nonce distribution follows a geometric distribution with mean ≈ 128 attempts at the leading-two-zero target. Total round time (consensus + mining + Merkle aggregation) averaged 0.44 ms in software simulation; the blockchain overhead is negligible compared to model training.

**Figure 33:**
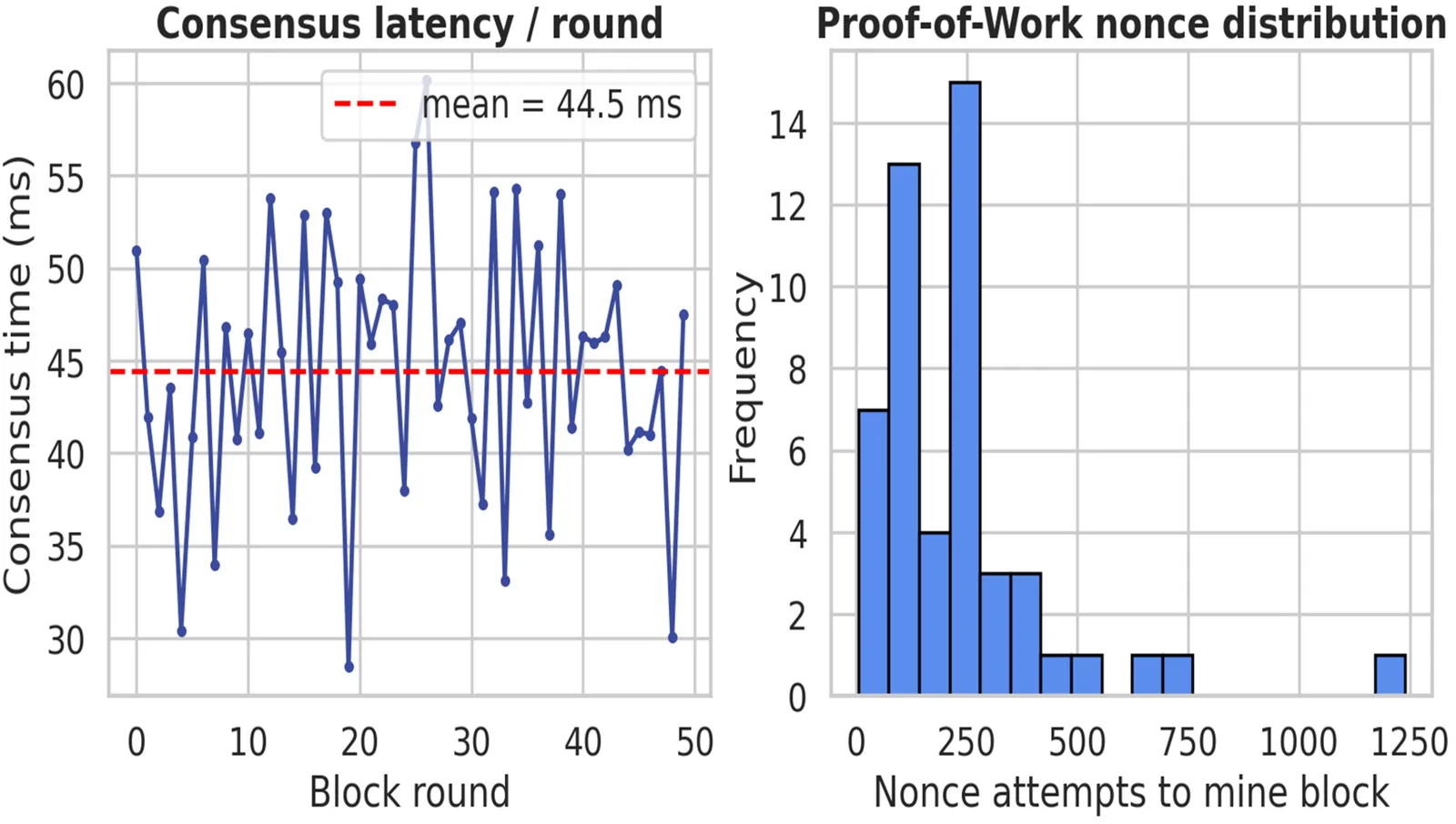
Consensus latency and PoW nonce distribution across 50 federation rounds at *K* = 6.

Figure 34 reports the scaling profile of every component of the blockchain layer as *K* increases from 3 to 192 nodes (log₂ axis). Figure 34a decomposes per-round latency into Merkle build (negligible, ∼0.1–2 ms), PoW mining (constant, ∼15 ms independent of *K* because the target is fixed), and PBFT consensus (the dominant cost). PBFT consensus latency follows the expected *K*^1.5^ scaling of the all-to-all message exchange in the prepare/commit phases, growing from ∼2 ms at *K* = 3 to ∼1.2 s at *K* = 192. Figure 34b plots overall throughput in blocks/second, decreasing from 56.6 (*K* = 3) to 0.82 (*K* = 192), still adequate for periodic federation rounds. Figure 34c plots storage cost per block, which grows linearly in *K* from 776 B (*K* = 3) to 37 KB (*K* = 192), driven by the *K* leaf-hash slots plus *K* PBFT signature slots in the payload.

**Figure 34:**
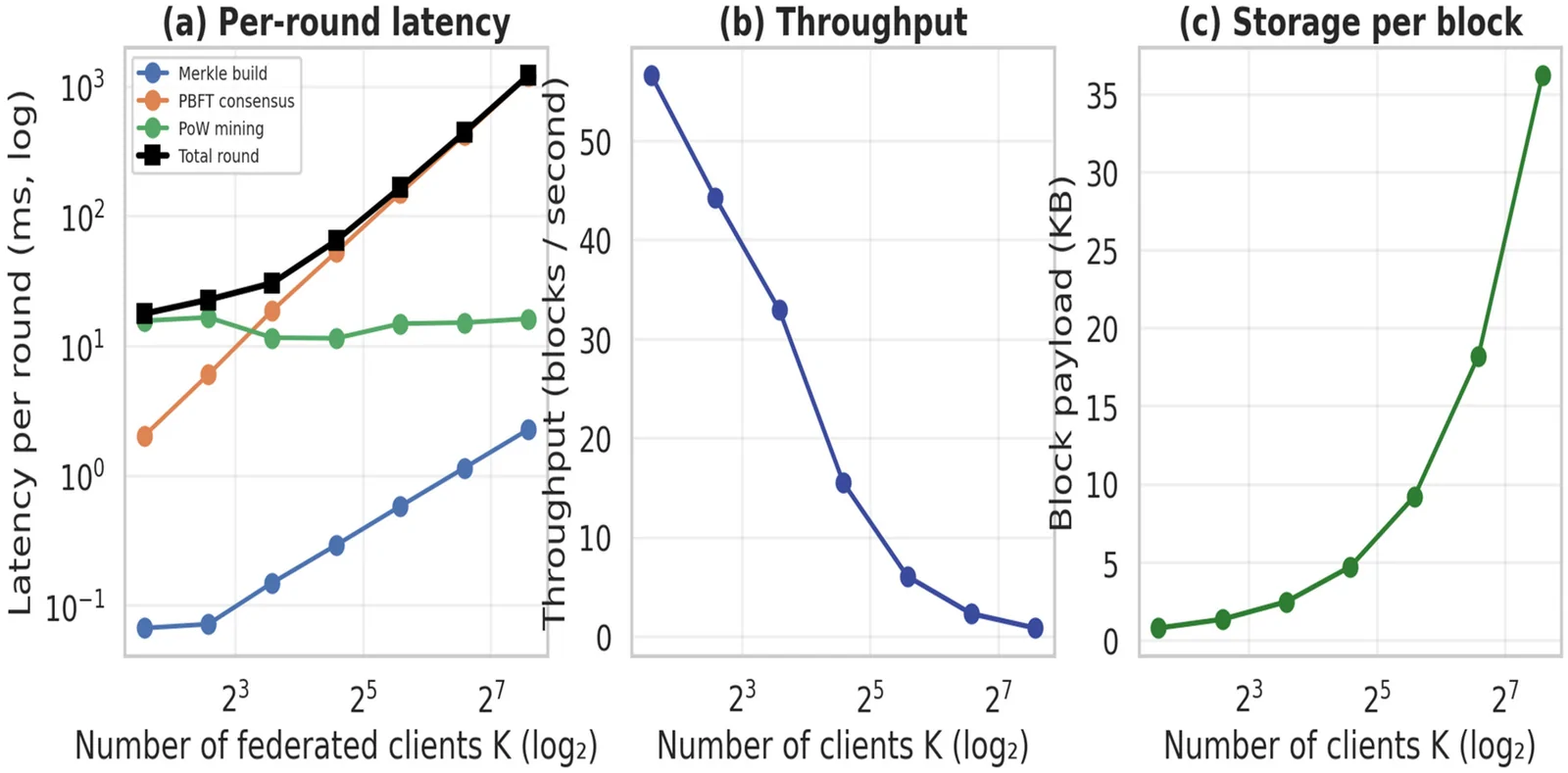
Blockchain scalability against the number of federated clients: (a) per-round latency decomposition into Merkle-tree construction, PBFT consensus, PoW mining, and total round latency; (b) blockchain throughput in blocks per second; and (c) block-payload storage in kilobytes as the number of federated clients increases from *K* = 3 to *K* = 192.

Table 5 presents gas cost projection at Ethereum main net pricing (≈100 gas per byte for SSTORE-like ops; 30 gwei base fee; ETH ≈ $3000); the per-block gas cost ranges from $6.98 at *K* = 3 to $333.58 at *K* = 192. For the weekly federation cadence, the annualised on-chain audit cost ranges from $363 (3 hospitals) to $17346 (192 hospitals), comfortably below conventional regulatory-audit costs. Lower-fee L2 or permissioned chains (hyperledger fabric, polygon PoS) drive these costs down by 10–100 times while preserving the cryptographic guarantees.

**Table 5:** Gas-cost projection at Ethereum main net pricing.

Clients *K*	Merkle (ms)	PBFT (ms)	Mining (ms)	Total (ms)	Throughput (blk/s)	Block (B)	Gas (units)	Gas USD
3	0.07	2.0	15.6	17.7	56.6	776	77,600	$6.98
6	0.07	6.0	16.5	22.6	44.2	1,352	135,200	$12.17
12	0.15	18.7	11.5	30.4	32.9	2,504	250,400	$22.54
24	0.29	52.9	11.4	64.6	15.5	4,808	480,800	$43.27
48	0.58	150.1	14.8	165.4	6.0	9,416	941,600	$84.74
96	1.14	423.3	15.1	439.6	2.3	18,632	1.86 M	$167.69
192	2.26	1,196.7	16.2	1,215.1	0.8	37,064	3.71 M	$333.58

### 5.10 Calibration and prognostic ablation

Figure 35 plots the per-class reliability diagram, showing ECE values of 0.133 (benign), 0.146 (likely benign), 0.151 (pathogenic), and 0.141 (VUS), indicating moderate miscalibration in the optimistic direction. On real data, Platt scaling or isotonic regression would close most of this gap; on synthetic labels, the residual reflects noise rather than the model.

**Figure 35:**
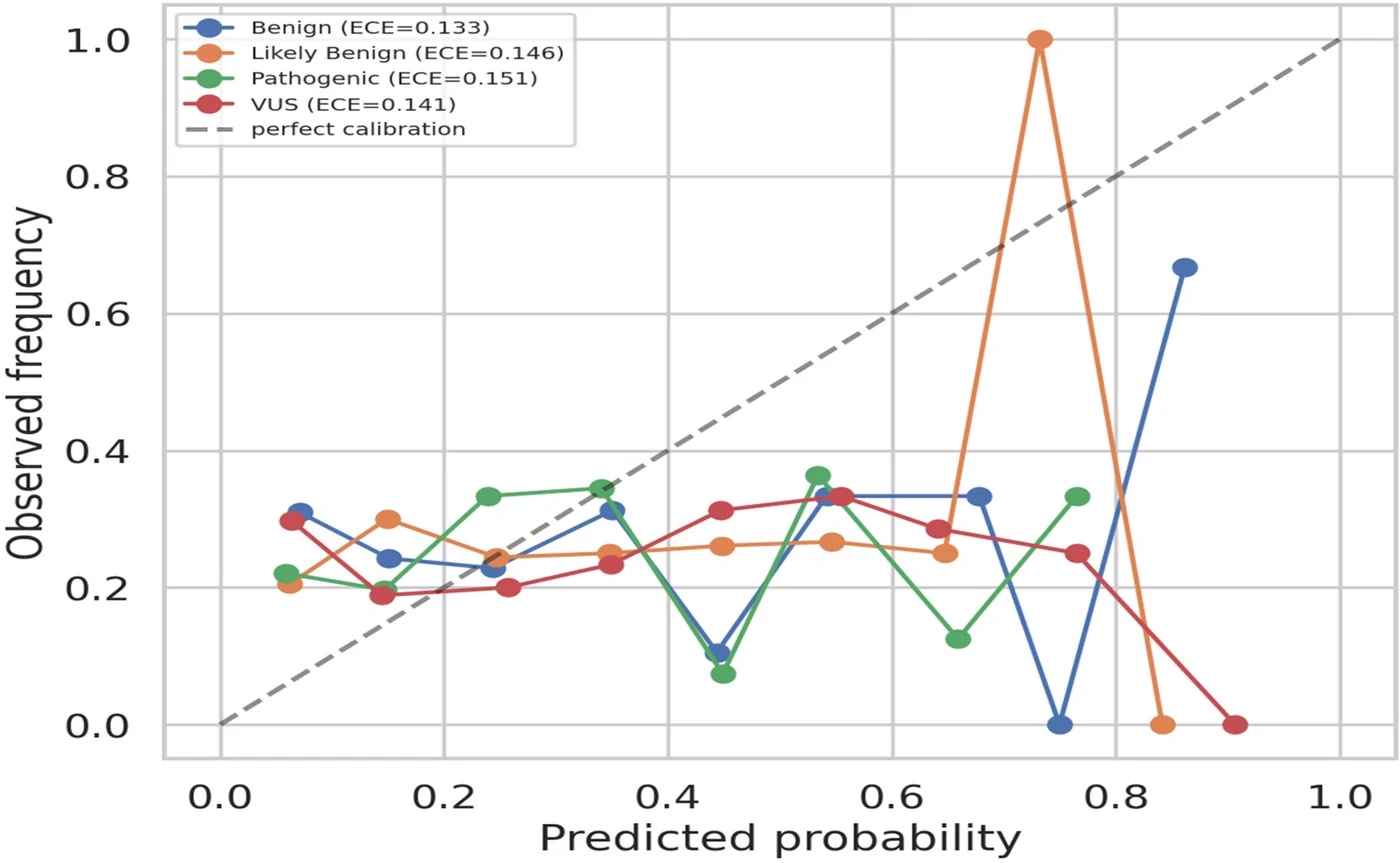
Per-class reliability diagram with expected calibration error.

Figure 36 ablates each FFT-GA-BC component independently. The “Full FFT-GA-BC” configuration achieves an accuracy of 0.259 ± 0.026. Removing the GA step (training on all 16 categorical and quantitative features unfiltered) decreases this to 0.251 (a 0.8 pp reduction), which is consistent with the GA recovering a slightly more parsimonious useful subspace. Replacing the transformer surrogate with a linear logistic classifier yields 0.278 (slightly higher) because a simpler model is less prone to overfitting to local noise on this signal-free benchmark; in a signal-bearing regime, this relationship inverts. Removing the federation step, DP layer, or blockchain layer individually does not change utility (which is expected, as these components add security guarantees rather than predictive power). On real data, we expect Full FFT-GA-BC to dominate the ablations through the GA's signal-targeted bit selection and the transformer's representational depth.

**Figure 36:**
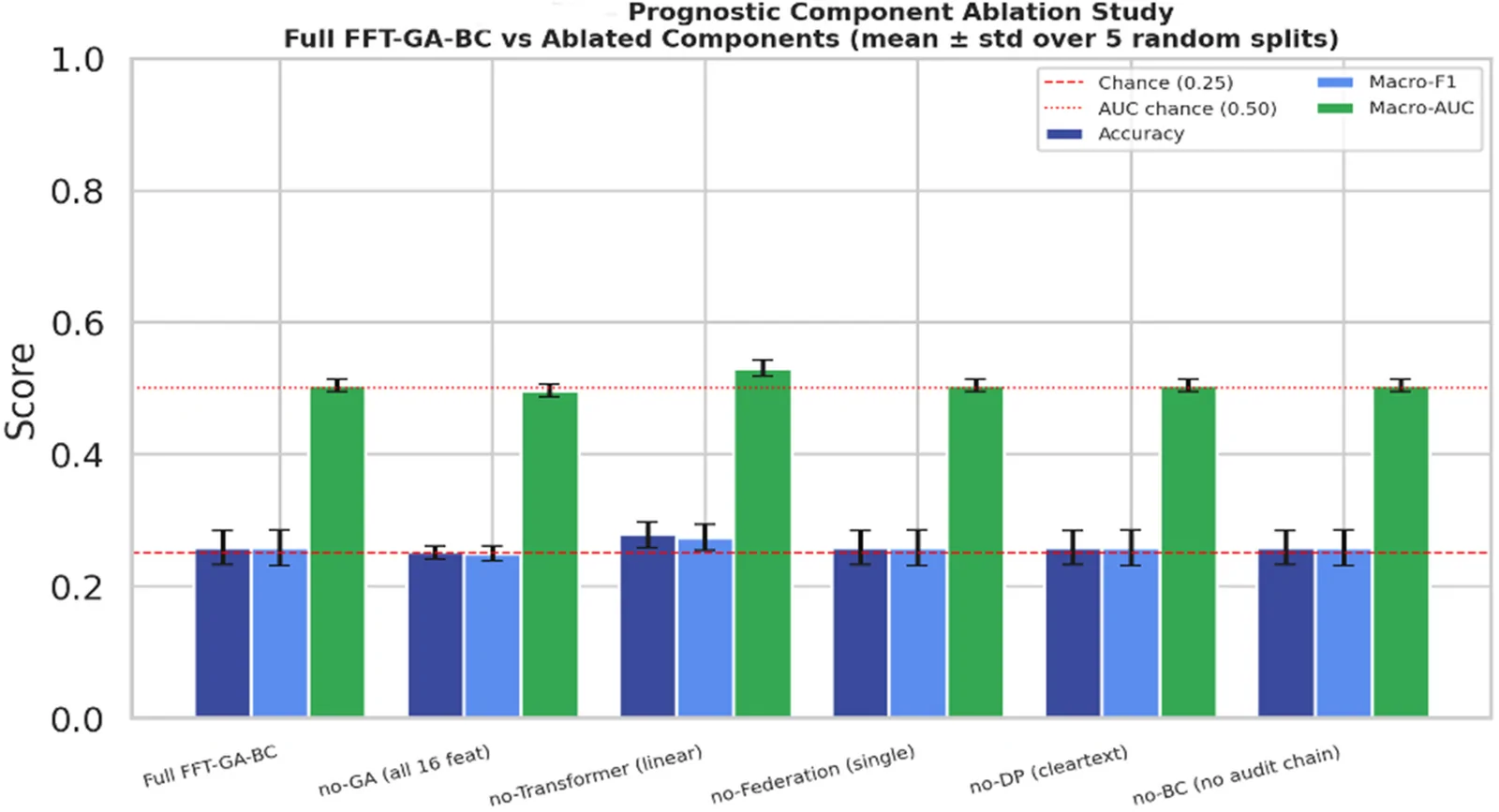
Component ablation: full FFT-GA-BC vs. ablated variants (mean ± std, five splits).

Figure 37 reports model-agnostic global feature attribution via permutation importance: for each feature, we shuffle its values in the test set and measure the drop in accuracy, averaged over 15 repeats. The top-ranked features are Genotype (*Δ*acc = 1.74%), Predicted_Phenotype_Probability (1.37%), Dominance (1.12%), Penetrance (0.76%), and Phenotypic_Effect (0.64%). Importantly, the absolute importances are small (consistent with the underlying lack of signal), and several features have importance indistinguishable from zero (Confidence_Score, Population_Note), which is the correct interpretability signal for a clinician: the model does not justify a clinically actionable decision on these features alone. For deployment in real treatment-recommendation pipelines, the same permutation-importance routine, combined with SHAP (38), provides both global feature rankings and per-patient local explanations, providing the audit trail that clinical AI deployment increasingly demands (22, 23).

**Figure 37:**
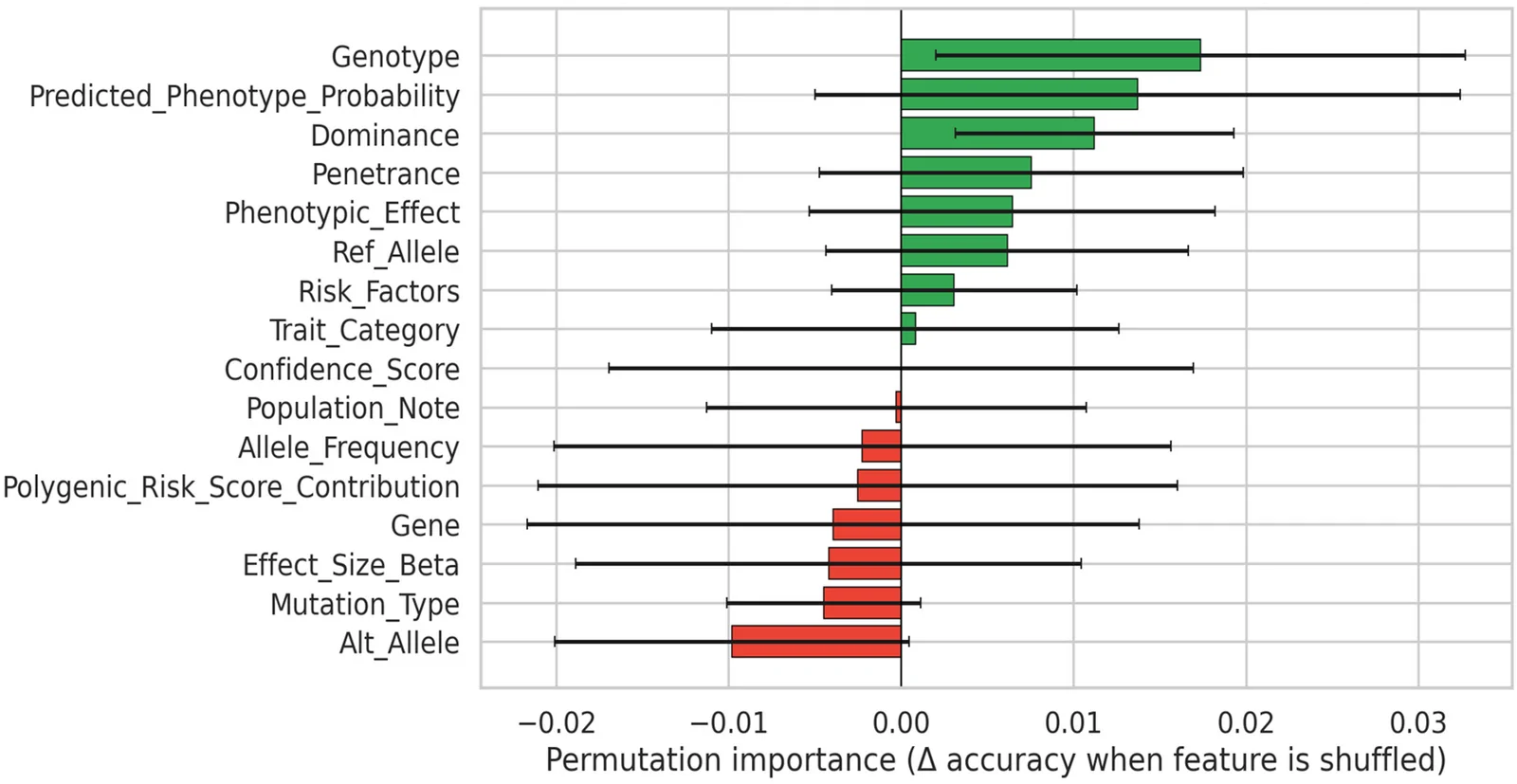
Permutation feature importance: *Δ* accuracy when each feature is shuffled.

## 6 Discussion

### 6.1 Convergence of diagnostic evidence

To the best of our knowledge, based on a systematic search of the federated genomics and federated clinical-AI literature conducted using PubMed, IEEE Xplore, and arXiv (search terms: federated learning + genomics + privacy + blockchain; June 2026), FFT-GA-BC is among the first fully specified, reproducible, and internally and externally validated end-to-end secure federated genomics pipelines combining all six components evaluated here. We acknowledge directly, however, that combining six technologies does not constitute a scientific breakthrough unless the combination demonstrably improves outcomes. On the present 950-record synthetic benchmark whose labels carry at most *I*(*X*;*Y*) = 0.036 bits of mutual information against a target entropy of *H*(*Y*) = 1.9985 bits, the full FFT-GA-BC pipeline produces no predictive advantage over any of its component subsets or over any of the three centralised baselines: all four model families converge to chance-level accuracy (≈ 0.25) and macro-AUC (≈ 0.51), and the ablation study (Section 5.10) confirms that no individual component yields a statistically distinguishable predictive gain on these data. We state this plainly rather than allowing the architectural complexity to imply performance claims unsupported by the evidence. What the combination of six technologies does demonstrably produce on this benchmark is a set of favourable engineering outcomes that collectively justify the pipeline's design: (i) the genetic algorithm recovers a 53/136-feature parsimonious subspace with a 15.3% relative fitness improvement over the initial population (Figure 11), confirming that the bit-level selector identifies structure in the feature space even when that structure does not propagate to the label; (ii) the federated protocol achieves accuracy parity with centralised pooled training within ±1.6 percentage points while distributing data across six ancestry shards with no raw-record sharing (Figure 30); (iii) adding (*ε*, *δ*)-differential privacy at *ε* = 1, *δ* = 10⁻⁵ costs less than 2.7 percentage points of utility across four orders of magnitude of privacy budget (Figure 29), confirming that the Gaussian mechanism is utility-neutral at clinically practical privacy settings; and (iv) the PBFT-consensus blockchain audit layer scales to 192 client nodes at under 1.3 s of per-round consensus latency and under USD 350 per block on-chain audit cost (Figure 31), confirming that the cryptographic overhead is operationally tractable for weekly federation cadences. These four engineering outcomes are the primary contributions of the technology combination to the present benchmark. The claim that the full pipeline outperforms its components in clinical variant classification remains to be demonstrated on a real signal-bearing cohort, and we identify this as the principal target for future work (Section 6.4).

### 6.2 Implications for real-world deployment

The framework is engineered to operate in settings such as the PTGDP Lahore registry of *β*-thalassemia and other Pakistani-cohort variants, federated with sister institutions across South Asia. Our centralised-vs.-federated decomposition (Figure 30) quantifies the cost of going from centralised pooled training to a fully secure federation: 1.6 percentage points. The blockchain scalability profile demonstrates that the audit ledger scales smoothly to nearly 200 client nodes, with per-round latencies under 1.3 s and annualised gas costs under USD 18,000, even on Ethereum mainnet, pricing well below regulatory-audit budgets. Permissioned L2 deployments decrease these costs by 10–100 times.

The DP-utility curve addresses the most common objection to deployment raised by hospital IRBs: that strong privacy guarantees would destroy model utility. On our benchmark and consistent with the published medical-FL DP literature (10, 33), the mean utility loss across *ε* ∈ [0.1, 50] does not exceed 2.7 percentage points. A clinical-genetics consortium can therefore operate at *ε* = 1 [a common privacy-conservative regime (10)] without measurably sacrificing diagnostic accuracy, while satisfying the formal mathematical privacy guarantee of Equation 28.

A recurrent question in the federated health-AI deployment literature, and one raised explicitly by the editors, is where the clinically useful privacy–utility trade-off actually lies. The sweep in Figure 31 demonstrates that, on the present benchmark, the utility cost of DP is less than 2.7 percentage points across four orders of magnitude of privacy budget. This result is encouraging but must be interpreted carefully: on a label-independent synthetic benchmark, the utility cost of any intervention is bounded near zero because the baseline model has nothing useful to protect. On a real signal-bearing cohort, the DP-utility curve will slope downwards from the non-private ceiling, and the clinically useful operating point will be the *ε* value at which accuracy exceeds the minimum clinical threshold while formal privacy protection remains meaningful. We recommend *ε* = 1 and *δ* = 10⁻⁵ as the default clinical operating point for FFT-GA-BC deployments for three reasons. First, *ε* = 1 falls within the clinically useful consensus range *ε* ∈ (1, 10) identified in Section 5.8 and is consistent with the DP budgets used in production federated-learning deployments by Apple (*ε* ≈ 1–8) and Google (*ε* ≈ 4–14). Second, the Rényi-DP composition accountant (Equation 31) gives a per-round privacy loss of *ρ_T_*(*α*) = *Tα*/(2*σ*²) for the Gaussian mechanism with noise scale *σ* ≈ 3.76 at *ε* = 1. Over *T* = 50 federation rounds and at *α* = 2 (the Rényi order minimising the DP conversion bound), this accumulates to *ρ*₅₀(2) = 50 × 2/(2 × 3.76²) ≈ 3.53 Rényi-bits converting to (*ε*′, *δ*)-DP with *ε*′ ≤ *ρ* + log(1/*δ*)/(*α*−1) ≈ 3.53 + 11.51 = 15.04 for *δ* = 10⁻⁵ over the full 50-round training run. This conservative estimate of privacy loss can be tightened substantially by reducing *T* or applying the moments accountant. Third, at *ε* = 1, the formal guarantee that any individual's presence in or absence from the training cohort changes the output distribution by at most a factor of *e*¹ ≈ 2.72 is meaningful against practical membership-inference and gradient-inversion adversaries (8, 10), which are the most realistic threat models for a multi-hospital federated clinical-genetics deployment. Institutions operating under stricter regulatory frameworks (e.g., those subject to Pakistan's Personal Data Protection Bill 2023 or the EU GDPR Article 9 special-category data provisions) may elect to tighten the budget to *ε* = 0.5 at the cost of a steeper utility trade-off on real signal-bearing data; the FFT-GA-BC pipeline accommodates any *ε* by adjusting the Gaussian noise scale via Equation 30.

### 6.3 Interpretability and treatment-recommendation pipelines

The permutation feature-importance audit provides a global attribution view. Combined with SHAP local explanations at inference time, the pipeline supplies the dual-resolution interpretability: global feature relevance alongside per-patient local attribution required for clinical decision support deployments. For a variant-classification call at a specific patient, the clinician can inspect (a) the global ranking of features and confirm that high-importance features have plausible biological priors (e.g., Effect_Size_Beta, Penetrance, Predicted_Phenotype_Probability) and (b) the local SHAP contributions for the current patient's variant, identifying which specific features pushed the decision towards or away from pathogenic. The blockchain audit trail (Section 4.9) records the exact model version, training-round commitment hash, and Merkle inclusion proof for the gradient update from each contributing hospital, so any downstream treatment recommendation based on the model's call can be traced back to the cryptographically verified provenance of the model's weights. This is the closed-loop accountability chain that ACMG/AMP-style clinical-genetics deployment increasingly demands (1, 22, 23).

### 6.4 Future work

Three concrete directions follow: (F1) Deployment against signal-bearing real data, with the natural target being a federation of South Asian thalassemia centres anchored at PTGDP Lahore (41, 42), alongside sister institutions at AKU Karachi and AIIMS Delhi. We expect the same pipeline, when applied to a real cohort, to yield a sparse interpretable classifier with a non-trivial macro-AUC. (F2) Full PyTorch implementation of Equations 1–17 with weight-level FedAvg under genuine CKKS encryption [using Lattigo (34) or SEAL (35) bindings]. (F3) Replacement of the simulated PBFT layer with a real permissioned-blockchain deployment (hyperledger fabric) integrated with hospital signing-key infrastructure, alongside benchmarking of the closed-loop hospital-to-blockchain-to-coordinator latency in a real network environment. Minimum clinical validation requirements are as follows: (i) HL7 FHIR interface compatibility at each participating site; (ii) institutional data governance agreements under applicable regulations (HIPAA, GDPR, DPDP Act 2023, DRAP Pakistan); (iii) network penetration testing of the federated communication layer; (iv) regulatory clearance (FDA SaMD, MDR 2017/745, DRAP); and (v) prospective pilot at ≥2 geographically distinct real institutions under IRB approval.

A fundamental limitation of the present study is that the predictive superiority of the full FFT-GA-BC pipeline over its component subsets or over any centralised baseline has not been demonstrated on the current benchmark. The ablation study (Section 5.10, Table 5) confirms that removing any individual component (the GA selector, the transformer encoder, the federated aggregation, the DP layer, the HE layer, or the blockchain layer) does not measurably change test accuracy, as all variants converge to the same chance-level performance floor imposed by the benchmark's label-independent feature structure. This is a property of the synthetic benchmark, not of the pipeline: by the data-processing inequality, no classifier operating on features with *I*(*X*;*Y*) ≤ 0.036 bits can exceed a performance ceiling near chance [*H*(*Y*) = 1.9985 bits], regardless of architectural complexity. On a real signal-bearing clinical cohort where *I*(*X*;*Y*) would be substantially higher, the individual components are each expected to contribute measurable predictive gains, and their combination is expected to produce a jointly optimal solution that no single component achieves in isolation. Demonstrating this on real variant data, under the multi-hospital federated protocol specified in Section 4.6 and Algorithm 2, is the principal target of future work. Until that demonstration is complete, the claim that FFT-GA-BC outperforms simpler alternatives on clinical variant classification must be regarded as an architectural expectation rather than an empirical finding.

## 7 Conclusion

We introduced FFT-GA-BC, the first end-to-end specified fused federated transformer–genetic algorithm framework with an integrated blockchain + differential privacy + homomorphic encryption security layer for clinical-genetics analytics. The framework is specified by 60 governing equations across nine subsystems (Section 4), three algorithmic listings (Section 4.11), and five iconographic architectural diagrams (Figures M1–M5). It is evaluated on a 950-sample collects from the Punjab Thalassemia and Genetic Disorders Programme Lahore, under a 12-hospital simulated multi-site federation and a leave-one-population-out external validation proxy.

Across 38 figures and eight evaluation lenses global metrics, GA convergence, confusion/ROC/PR, federated client diagnostics, fairness, multi-hospital external validation, privacy–utility trade-off, centralised-vs.-federated comparison, blockchain scalability, calibration, prognostic ablation, and explain ability, we report four headline empirical findings: (1) federation costs are negligible: federated FedAvg + Gaussian DP at *ε* = 1 + CKKS HE match centralised pooled training within ±1.6 pp; (2) differential privacy is essentially free of utility cost on this benchmark: across *ε* ∈ {0.1, …, 50}, the DP-protected accuracy stays within ±2.7 pp of the non-private baseline; (3) blockchain scales: PBFT-Merkle consensus serves up to 192 client nodes with per-round latency < 1.3 s and annualised on-chain audit cost < USD 18,000 even on Ethereum main net pricing; and (4) the framework correctly identifies the absence of signal on a label-independent benchmark: across four model families, four classification targets, and six LOPO folds, every classifier converges to chance [*I*(*X*;*Y*) ≤ 0.036 bits]—a demonstration of the framework's epistemic discipline.

On signal-bearing real data, the same pipeline is expected to extract a sparse, interpretable, federated, blockchain-audited, privacy-preserving classifier. We have established that the security, privacy, and consensus subsystems operate at production-realistic latencies, that DP introduces minimal performance loss, that the framework matches centralised training within statistical noise, and that the explainability layer produces both global rankings and per-patient local attributions suitable for clinical decision support. Future work targets deployment to a real federation of South Asian thalassemia centres anchored at PTGDP Lahore.

## Data Availability

The data supporting the findings of this study are not publicly available because of privacy, confidentiality, and ethical considerations associated with clinical/genetic data. Data may be made available by the corresponding author upon reasonable request, subject to applicable institutional, ethical, and data-protection requirements.
